# Immobilization of Metal Hexacyanoferrate Ion-Exchangers for the Synthesis of Metal Ion Sorbents—A Mini-Review

**DOI:** 10.3390/molecules201119718

**Published:** 2015-11-19

**Authors:** Thierry Vincent, Chloë Vincent, Eric Guibal

**Affiliations:** Ecole des mines d’Alès, Centre des Matériaux des Mines d’Alès, 6, avenue de Clavières, F-30319 ALES cedex, France; thierry.vincent@mines-ales.fr (T.V.); chloe.vincent@mines-ales.fr (C.V.)

**Keywords:** metal hexacyanoferrate, ion-exchanger, composite materials, *in situ* synthesis, encapsulation, polymers, biopolymers, porous mineral supports

## Abstract

Metal hexacyanoferrates are very efficient sorbents for the recovery of alkali and base metal ions (including radionuclides such as Cs). Generally produced by the direct reaction of metal salts with potassium hexacyanoferrate (the precursors), they are characterized by ion-exchange and structural properties that make then particularly selective for Cs(I), Rb(I) and Tl(I) recovery (based on their hydrated ionic radius consistent with the size of the ion-exchanger cage), though they can bind also base metals. The major drawback of these materials is associated to their nanometer or micrometer size that makes them difficult to recover in large-size continuous systems. For this reason many techniques have been designed for immobilizing these ion-exchangers in suitable matrices that can be organic (mainly polymers and biopolymers) or inorganic (mineral supports), carbon-based matrices. This immobilization may proceed by *in situ* synthesis or by entrapment/encapsulation. This mini-review reports some examples of hybrid materials synthesized for the immobilization of metal hexacyanoferrate, the different conditionings of these composite materials and, briefly, the parameters to take into account for their optimal design and facilitated use.

## 1. Introduction

The development of the nuclear industry has generated a number of different effluents containing radionuclides, long- or short-life elements, issued from normal running conditions or from incidental events such as Three Mile Island (USA), Chernobyl (Ukraine) or Fukushima (Japan). There is a need for materials and/or processes capable of recovering radionuclides from low metal concentration solutions containing, in some cases, high salt levels (sea water for example, or alkaline solutions, such as the Hanford Site tanks).

Different processes have been developed for the liquid extraction, the precipitation (or co-precipitation), or the sorption of radionuclides from such complex media. Mineral ion-exchangers have shown their high efficiency for the recovery of radioelements, based on their crystalline structure, which contributed to sieving the target metals from other contaminants. Among ion-exchangers Prussian-Blue analogues (double ferrocyanides or hexacyanoferrates) have retained a great attention, and they are frequently used for Cs recovery from industrial effluents, contaminated water bodies [1], and for Cs or Tl decorporation [2,3,4] (the so-called commercial Radiogardase^®^ [5]). Transition metal hexacyanoferrates are very efficient and selective ion-exchangers for Cs^+^ due to their cubic structure with thin channels (close to 3.2 Å) [6]: this size is compatible with the diffusion of small hydrated ions like Cs^+^, K^+^, NH_4_^+^ while the diffusion is hindered for metal ions with largest hydrated radius such as Na^+^, Ca^2+^, *etc.* The recovery of target metal can also proceed through a reactive precipitation involving the simultaneous binding of the radionuclide and the formation of the metal hexacyanoferrate [7,8]: the precursors (alkali metal ferrocyanide and metal salt) are mixed with cesium ions. However, the very thin precipitates formed during the reaction may be difficult to separate.

The sorption performance of these materials is generally controlled by diffusion, accessibility to reactive sites and surface parameters. This means that these ion-exchangers exhibit their highest efficiency for sub-micron size particles. However, designing and managing such materials at the “nanometer” scale leads to complex problems for their management including complex recovery of materials at the end of processing flow, possible dispersion of hazardous materials (nanometer size particles, radionuclide-bearing compounds). These management and confinement issues are significantly limiting the dissemination of the technique and require developing sophisticated systems for management of these fine particles.

One solution to overcome these problems consists in immobilizing the active material (ion-exchanger micro/nanoparticles) at the surface or in the core of supports to prevent their readily dispersion. However, the immobilization process may face some limitations, including: reduced reactivity, low volumetric/mass density of reactive sites (dilution of active particles in/on the matrix) or slow kinetics (increased resistance to diffusion). Thus the main question is: “How to handle simultaneously confinement issues, mass transfer properties and high volumetric density of reactive groups?”

This review reports on: (a) the synthesis and characterization of bulk hexacyanoferrate-based ion-exchangers; (b) the techniques that can be used for their immobilization on different supports (including the possibilities offered for adapting the shape and the morphology of these hybrid materials); and briefly (c) their sorption properties.

We note that though most of the review focuses on hexacyanoferrate(II) (or ferrocyanide) of bi-metal complexes (*i.e.*, K^+^/Na^+^/NH_4_^+^ and divalent counter cation), some references dealing with simple hexacyanoferrate(II) or hexacyanoferrate(III) will be discussed for the purpose of commenting on some of the parameters affecting metal binding.

Hexacyanoferrate ion-exchangers have been widely studied for the recovery of Cs(I) and analogues; however, a numerous literature is also citing the possibility to use these materials for the sorption of base and precious metal ions. This mini-review is also reporting some examples of synthesis of hexacyanoferrate composite materials for the recovery of non-radioelements (*i.e.*, Tl(I) and base/precious metal ions).

## 2. Synthesis and Sorption Properties of Bulk Hexacyanoferrate-Based Ion-Exchangers

The structural properties and sorption performances of hexacyanoferrate-based ion exchangers are strongly influenced by the composition of ion-exchangers (types and contents of monovalent cation and multivalent cation), which depends on the conditions used for their synthesis.

### 2.1. Synthesis and Structure of Metal Hexacyanoferrate Ion-Exchangers

**Figure 1:**
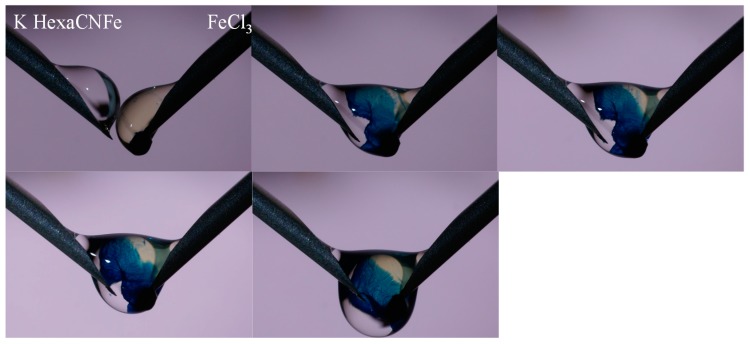
“In-drop” synthesis of Prussian Blue.

Basically, the synthesis of insoluble hexacyanoferrate compounds proceeds through the reactive precipitation of two precursors: a soluble hexacyanoferrate (based on K, Na, H or NH_4_^+^ forms) and a soluble metal salts (to be incorporated in the double-metal hexacyanoferrate compound). The experimental procedure influences the properties of the final product; including the solubility/stability, the composition (ratio metal/K^+^, Na^+^, H^+^ or NH_4_^+^), the size of nano/micro-particles (and their aggregation), the specific surface area, the density and the crystalline structure (face-centered cubic, rhombic, cubic, rhombohedric, trigonal) and its cell parameter [9,10,11]. The parameters that play on the final product are the types of precursors, their molar ratio (and their concentrations), the mode (simultaneous introduction, successive addition, *etc.*), the order (hexacyanoferrate into metal salt solution, or reciprocal) and velocity of introduction (drop by drop, slow, fast, *etc.*) of their addition, the reaction temperature, the maturation (or aging), and the post-treatment (drying conditions) [12]. This means that slightly changing the experimental conditions may significantly change the characteristics of the final product. This also means that during the synthesis of the double hexacyanoferrate compound a variation in experimental conditions may cause (a) the co-existence of different products, and (b) the difficulty in obtaining perfectly reproducible results. The molar ratio during the synthesis procedure may change when one of the precursors is injected drop to drop in the other compound, driving to heterogeneous materials. This drawback can be minimized with pouring out together the two precursors at the same moment. However, some heterogeneity can locally occur in the mixing zone of the two compounds. An alternative solution would consist in the “in-drop” synthesis of the double-metal hexacyanoferrate. The process consists in the mixing of the two precursors in a single drop through two needles fed at the same rate by a peristaltic pump. Figure 1 shows the example of the synthesis of Prussian Blue by this “in-drop” method: the two precursors (iron(III) chloride and potassium hexacyanoferrate) are mixed in the drop at the extremity of the needles (the photographs taken at different contact times show the progressive formation of the Prussian Blue complex, together with the beige colored phase of FeCl_3_ and the uncolored phase of potassium hexacyanoferrate).

Despite a numerous literature on hexacyanoferrate use for Cs (and other radionuclide) binding, this is generally difficult getting full information on the synthesis procedure and detailed characteristics of the products (structure, composition, surface properties, and comparative sorption properties) [13]. As a consequence it is difficult to correlate the optimum conditions for sorption of target radionuclides with the synthesis procedure and the properties of double-metal hexacyanoferrate ion-exchangers. Exhaustive reviews on the preparation and characterization of metal hexacyanoferrate (Cu, Zn and Ni hexacyanoferrates) have been published in the 80’s and 90’s by Loos-Neskovic research group: they reported and discussed the impact of synthesis procedures on the structure of double-metal hexacyanoferrate [11,14,15]. Exhaustive structural characterization of these bulk ion-exchangers have been reported in this “reference literature” and readers are invited to consult these papers for literature analysis. The chemical structure of the Prussian Blue analogue is characterized by the general structural formula: A_x_M_y_[Fe(CN)_6_]_z_·nH_2_O, with the specific *x*, *y*, *z* and *n* parameters and the type of monovalent cation (A = K^+^, Na^+^, NH_4_^+^), and bivalent transition metal cation (M = Ni^2+^, Co^2+^, Cu^2+^, Zn^2+^, Fe^2+^, *etc.*). This general chemical structure, the physical structure (cubic, face-centered cubic., trigonal, tetragonal, rhombohedral), and the size of crystallites are strongly controlled by the experimental conditions used for the synthesis of the ion-exchanger [15]. Loos-Neskovic group showed that the most important and controlling parameters are the molar ratio between the precursors, the order of their addition, the presence of other chemical agents (ammonia and ammonium chloride, *etc.*), the temperature, the pH. These parameters may impact also the stability of intermediary colloid particles and finally the size of ion-exchanger particles [15]. This is critical for the sorption properties of Prussian Blue analogue: hence, Lee and Streat [16] reported that Cs sorption capacity of potassium cobalt ferrocyanide is directly correlated to the fraction of potassium present on the ion-exchanger. It is noteworthy that the synthesis procedure may also impact the oxidation state of iron (Fe(II) *vs.* Fe(III)) in the ion exchanger: the change in the precursor (ferricyanide *vs.* ferrocyanide) leads to different arrangements in the mesh of the crystalline structure, the vacancy of ferro-/ferri-cyanide sites, the size of ion-exchange cage, the possible incorporation of H_2_O molecules (which, in turn, may affect the coordination sphere and the reactivity of cyanide groups).

The type of metal may significantly impact the structure and the size of double-metal hexacyanoferrate crystals. For example, Loos-Neskovic *et al.* [17] reported that Zn-hexacyanoferrate(III) crystals had trigonal structure at high Zn content and a rhombohedral structure at lower Zn content, while nickel-hexacyanoferrate(III) crystals exhibited a face-centered cubic structure. Table S1 (see Supplementary Materials) compares the characteristics of different metal-potassium hexacyanoferrates encapsulated into chitin beads. The ion-exchanger content in the different composites was of the same order (*i.e.*, between 16% and 20%, *w*/*w*); the amount of K element in the composites strongly varied indicating significantly different possibilities of ion-exchange (*i.e.*, between 0.2 and 0.9 mmol·K·g^−1^ composite). However, the sorption capacities for Cs(I) were not directly correlated to the amount of K element (for both “natural” Cs(I) and ^137^Cs-doped solution) [18].

The ratio of precursors directly impacts the stability of the colloidal phase (formed during the reaction of the precursors) and the size of synthesized particles but hardly changes the type of produced hexacyanoferrate [15]. It is generally accepted that an excess of counter metal ion (secondary metal salt containing d-metals such as Cu, Zn, Co, Fe, Ni, among the most popular) contributes to reduce the tendency of the mixture to form stable colloids and to facilitate the precipitation of the complex. Loos-Neskovic *et al.* [14] compared the structure of zinc hexacyanoferrate(III) for different preparation methods with different molar ratio between the precursors (different excess of counter-metal): they most frequently found the structure to be rhombohedral but in some cases (and more specifically in the case of the presence of other cation like protons or Cs(I)) the structure changed to cubic. In addition, the chemical structure/composition was strongly affected by the experimental conditions that lead to mixtures of different salts and some changes in the crystal lattice. Figure S1 (see Supplementary Materials) shows the XRD patterns of Prussian Blue prepared by varying the molar ratio R between the precursors (R = [FeCl_3_]/[K_4_Fe(CN)_6_]). The XRD data were used for the determination of the size of sorbent particles (nanocrystals) through the Scherrer equation (Table S2, see Supplementary Materials). The size of nanocrystals was determined using 3 different peaks (at 2θ = 17.42, 24.63 and 35.22) corresponding to reflections (200), (220) and (400): the three determinations were roughly convergent in the range 58–82 Å, with dispersion lower than 8 Å (except for R: 1, for which the dispersion of particle size data was very large; *i.e.*, 103 ± 32 Å). The SEM-EDX analysis of the samples (compressed as discs) allowed determining the effective molar ratio Fe/K in the final products (Table S3, see Supplementary Materials). As expected increasing the ratio R increased the molar ratio Fe/K. Mimura *et al.* [19] investigated more specifically the impact of Ni/Fe molar ratio of precursors on the structure of potassium nickel hexacyanoferrate: as the Ni/Fe ratio decreased, the molar ratio K/Fe increased (while the theoretical ion-exchange capacity slightly decreased between 5.93 and 5.74 meq·g^−1^). The highest crystallinities and largest crystal sizes were obtained with K/Fe ratio of 1.15 and 1.32 (corresponding to molar excess of Ni *vs.* Fe in the precursors, in the range Ni/Fe: 1.43–4.0). Actually the comparison of maximum sorption capacities did not show clear and continuous trend: the sorption capacity varied between 1.0 and 1.5 mmol Cs·g^−1^.

The aging of the colloidal preparation (reaction of precursors, when the stoichiometry does not lead to the formation of a stable precipitate) is another way to control and increase the size of ion-exchanger particles, but the reproducibility in the synthesis procedure is more difficult to achieve. Loos-Neskovic *et al.* [15] also reported the possible effect of the temperature and drying on the structure and composition of the ion-exchanger. Ishfaq *et al.* [20] reported that increasing the drying temperature of potassium copper nickel hexacyanoferrate increases the Cs sorption capacity of the ion-exchanger: fast water release in the ion-exchanger enhances the porous properties of the material. Increasing the drying temperature from 70 to 110 °C leads to progressive increase of surface area (from 88 to 106 m^2^·g^−1^), pore size (from 60 to 85 Å), porous volume (from 0.26 to 0.51 cm^3^·g^−1^), capillary percentage (from 10% to 23%), and exchange capacity (from 1.39 to 2.25 meq·g^−1^). The expansion of surface properties with drying temperature was attributed to the faster evacuation of water molecules that contributes to the creation of supplementary pores. Gaffar *et al.* [21] reported the progressive change in the structure of potassium hexacyanoferrate crystals (space and lattice parameters) when increasing the temperature from 50 °C to 420 °C. The changes in the structure (tetragonal *vs.* monoclinic) were correlated to the progressive loss of crystallization water and the conversion of iron from ferrous form to ferric form (at the highest temperatures above 360 °C).

The presence of stabilizer during the synthesis also influences the size of particles. For example adding polyvinyl alcohol in the mixture of copper sulfate and potassium hexacyanoferrate allowed decreasing the size of copper hexacyanoferrate nanoparticles from 450 nm to 145 nm [22]. Similar conclusions were reached for the preparation of potassium-cobalt hexacyanoferrate ion-exchangers [23]. Another mode of synthesis may consist in the ion-exchange of a specific metal ion with an already existing solid hexacyanoferrate [24]. This mode of synthesis is less common and it is generally controlled by the exchangeability of the metal ion present on the solid ion-exchanger (depending on the difference in the affinity of metal ions with hexacyanoferrate moiety) [11,13].

### 2.2. Type of Metal Hexacyanoferrate

The chemical structure of the ion-exchanger obviously depends on the nature of the precursors used for the synthesis:

(a) Ferrocyanide and ferricyanide will influence the oxidation state of iron in the ion-exchanger [13,25], in addition to the effect of experimental conditions that may influence the stability and the oxidation/reduction of iron.
(b) The type of ferro/ferricyanide and more specifically the presence and the type of exchangeable alkali or monovalent cation (Na^+^, K^+^ or NH_4_^+^) will orientate the use of the ion-exchanger: for example, for Cs or Tl decorporation, potassium and ammonium salts should be prohibited to limit the secondary health effects and sodium-precursor will be preferred [26,27]. This may also influence the type of mechanism involved in metal binding: for example, the presence of monovalent cation allows ion-exchange process (instead of pure surface sorption) [25].
(c) The type of counter metal salt (nickel, copper, cobalt, iron, zinc) will influence the potential release of counter metal cations during the sorption process [18], but also the chemical structure [28], and the spatial arrangement (crystallographic properties) of the ion-exchanger, which, in turn, may affect the accessibility and the ion-exchange affinity (cage effect) of the material for target metal ions, but also uptake kinetics [25,29].

Table S4 (see Supplementary Materials) displays the different counter metals that were used for the synthesis of sorbents made of Prussian Blue analogues (including both precipitated particles and immobilized ion-exchangers). The most conventional hexacyanoferrates are incorporating: nickel [6,7,8,12,28,30,31,32,33,34,35,36,37,38,39,40,41,42,43,44,45,46,47,48,49,50,51,52,53,54,55,56,57,58,59,60,61,62,63,64,65,66], copper [8,11,13,22,25,28,30,31,38,42,45,46,47,48,49,58,61,64,65,67,68,69,70,71,72,73,74,75,76,77,78,79,80,81], iron [26,27,28,42,61,79,81,82,83,84,85,86,87,88,89,90,91,92,93,94,95,96], cobalt [1,8,9,23,28,30,31,42,45,46,47,48,58,75,88,97,98,99,100,101,102,103,104,105], zinc [14,45,46,47,48,50,106,107,108]. Other metals, more “exotic” in this field, have also been used for specific applications: de Taconi *et al.* [28] used Pd, In and V salts for the synthesis of metal hexacyanoferrates and reported their possible utilization in electrocatalytic devices (smart display windows, photoimaging, chemical/biochemical sensing, energy conversion, magnetic and optic materials, and environmental remediation).

**Figure 2:**
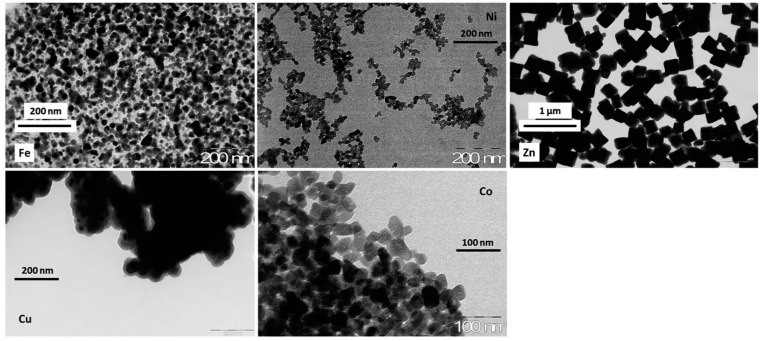
TEM photographs of bulk particles of metal hexacyanoferrates (reprinted with permission from Royal Society of Chemistry [18]).

Figure 2 compares the TEM photographs of bulk particles of metal hexacyanoferrates that were obtained using the synthesis procedure: reaction under strong agitation of two precursors (potassium hexacyanoferrate and metal sulfate for nickel, cobalt, zinc and copper, and chloride salt for iron(III), for Prussian Blue; *i.e.*, iron(III) complex the addition of the hexacyanoferrate was proceeded drop by drop). The TEM shows that all the ion-exchanger nanoparticles were smoothened, partially aggregated and small in size except for zinc ion-exchanger, where large isolated cubic particles have been identified. These materials have been immobilized in chitin beads [18]: the sorption properties of the composite materials have been compared. The maximum sorption properties are reported in Table S1). The sorbents can be ranked according: Cu > Ni > Zn > Co > Fe considering the sorption capacity of composite materials; however, reporting the sorption capacity as a function de actual content of the ion-exchanger in the composite material the sorbents can be ranked according: Cu > Zn > Ni > Co > Fe. It is not possible to find a direct correlation between the size of bulk ion-exchanger particles (Figure 2) and Cs(I) sorption capacities. In the case of bulk metal hexacyanoferrate, Grandjean *et al.* [29] reported a different order in Cs(I) sorption capacities (*i.e.*, Cu > Co > Ni) for cation free M^II^-Fe^III^ hexacyanoferrate. In addition, they observed that metal binding is controlled by a surface sorption mechanism limited by the surface of grain size. The best results obtained with copper hexacyanoferrate were attributed to the co-existence of ferro- and ferri-cyanides; indeed ferricyanide allows faster sorption and higher capacity as already reported by Ayrault *et al.* [25]. It is noteworthy that cesium sorption was systematically followed by the reduction of iron(III). They also reported that the presence of alkali ions (K^+^) in the framework of the ion exchanger was correlated to the enhancement of uptake kinetics and the increase of sorption capacities: this was attributed to grain size effect and to a change in the binding mechanism (ion exchange of potassium with cesium *vs.* surface sorption) [25,29].

### 2.3. Target Metals for Ion-Exchange on Hexacyanoferrate-Based Sorbents

Hexacyanoferrate-based sorbents are mainly oriented toward the recovery of Cs(I) [1,7,8,12,17,22,23,27,30,31,32,33,34,36,37,38,43,45,46,47,51,52,53,55,56,57,58,62,63,65,67,68,69,70,71,74,75,77,78,79,80,81,83,86,87,89,92,93,94,96,98,99,100,103,108,109,110,111,112,113,114,115,116,117,118,119,120,121,122,123,124,125,126]. However, some materials have been designed for the recovery of other alkali or alkaline-earth metals (including Rb(I) [54,116,120], and Sr(II) [43,91,107,123,127]) for radionuclide removal, and also for other metals that can be found in the effluents of nuclear industry or other industrial wastewaters such as Tl(I) [95], Co(II) [39,54,107,128], Au(III) [129], Cr(III) or Cr(VI) [44,54], As(III) [44], Pd(II) [49,130], Ag(I) [50], Pb(II) [73], Li(I) [116,120], I(I) [64], La(III) [107], U(VI) [116], Sb(III) [54] or Cu(II) [131]. Table S5 summarizes relevant literature for metal recovery on hexacyanoferrate-based sorbents (some references have been included though they are reporting simple interactions of hexacyanoferrate-based materials for the elaboration of advanced composites).

### 2.4. Binding Mechanisms

Based on the structure of these materials the main mechanism involved in the binding of Cs^+^ (and other similar elements) consists in the ion-exchange of K^+^ (or Na^+^, NH_4_^+^) for sorbents of general structure like A_x_M_y_[Fe(CN)_6_]_z_·nH_2_O (where A is the monovalent cation (K^+^, Na^+^, NH_4_^+^), M is the bivalent transition metal cation (such as Ni^2+^, Co^2+^, Cu^2+^, Zn^2+^, Fe^2+^, *etc.*)). Ramaswamy [58] investigated Cs^+^ sorption on a series of hexacyanoferrate(II)-based materials. The log-log plot of distribution coefficient against ammonium nitrate concentration gave straight lines with a slope close to 1 that confirm the 1:1 ion exchange mechanism between Cs^+^ and NH_4_^+^. However, this mechanism may be very complex and strongly depends on the structure (and the synthesis mode) of the ion exchanger as reported by Rykov *et al.* [132] in the case of hexacyanocobaltates (by analogy and probed by Mössbauer spectroscopy). Loos-Neskovic *et al.* [17] reported that for mixed hexacyanoferrate (K, Zn) Cs^+^ binding proceeds through the ion-exchange of K^+^ with Cs^+^ (and no Zn release) while for single zinc hexacyanoferrate, Zn^2+^ is released (following a non-stoichiometric ratio because of the partial binding of cesium through the sorption of ion pairs, as Cs^+^·Cl^−^). These different mechanisms are accompanied by possible changes in the crystalline structure of the materials (due to divalent d-metal release from crystal structure). Sheha [60] also reported several mechanisms for cesium recovery using potassium zinc hexacyanoferrate (deposited on magnetite) depending, in this case, on the pH of the solution. In neutral solutions Cs^+^ ions are exchanged with K^+^, while in acidic solutions a phase transformation occurred: potassium release due to proton exchange is followed by the formation of CsZn_2_Fe(CN)_6_ (instead of Cs_2_Zn_3_[Fe(CN)_6_]_2_ in neutral solutions with other phases being also present). Ofomaja *et al.* [133] incorporated iron(III) hexacyanoferrate on pine cone powder and investigated the sorption properties of the composite material for Cs^+^. After complete saturation of the sorbent with ammonium ions the NH_4_^+^-loaded composite is exchanged with Cs^+^, Na^+^ and Ca^2+^ solutions, separately. Ammonium ions are effectively released from the sorbent at different extent, according the ion-exchange facilities offered by the similarity in hydrated ionic radii between the different ions (*i.e.*, NH_4_^+^: 0.331 nm, Cs^+^: 0.329 nm, Na^+^: 0.358 nm, Ca^2+^: 0.412 nm [134]): Cs^+^ > Na^+^ > Ca^2+^. Based on these observations, Rb^+^ and Tl^+^ ions that have very similar hydrated ionic radii (0.329 and 0.330 nm, respectively) can be readily exchanged with potassium or ammonium ions. This can also participate to the efficient “sieving” effect of the zeolitic cage associated to the crystal structure of the ion-exchangers; this contributes to the high selectivity of these materials for the recovery of Cs-type radionuclides from complex solutions (such as found in high level wastes, HLW, or seawater).

Ayrault *et al.* [25] described the synthesis of “pure phase” copper ferrocyanide and copper ferricyanide that do not contain monovalent cation (like Cu^II^_2_Fe^II^(CN)_6_·xH_2_O and Cu^II^_3_[Fe^III^(CN)_6_]_2_·xH_2_O). They were produced by precipitation of precursors: copper(II) nitrate with sodium hexacyanoferrate(II) and potassium hexacyanoferrate(III), respectively. In the case of these ion-exchangers they reported complex mechanisms involving in a first step the diffusion of ions pairs (*i.e.*, Cs^+^·NO_3_^−^), followed by the reorganization of the solid (and concomitant formation of new solid phases) and partial release of copper. This mechanism is possible due to the presence of iron vacancies in the structure and to water replacing. The comparison of the two sorbents shows that Cu^II^_2_Fe^II^(CN)_6_·xH_2_O ion-exchanger has a much higher efficiency for Cs removal than Cu^II^_3_[Fe^III^(CN)_6_]_2_·xH_2_O; this was attributed to the presence of “free” copper (*i.e.*, not bound to CN) in Cu^II^_2_Fe^II^(CN)_6_·xH_2_O that allows the diffusion of ion-pairs. In the case of copper ferrocyanide (*i.e.*, Cu_2_Fe(CN)_6_·7H_2_O) Han *et al.* [70] also proposed an exchange of Cs^+^ with Cu^2+^ in the framework of the ion-exchanger. Qing *et al.* [135] correlated the selectivity of potassium-nickel hexacyanoferrate to hydrated radius: the distribution coefficient in the presence of equimolar concentrations of interfering cations (including NH_4_^+^, K^+^, Na^+^, Ca^2+^ and Mg^2+^) increases with the size of their hydrated radius; the cations having the closer hydrated radius from Cs^+^ have a stronger impact on its recovery.

Actually, the synthesis of Prussian Blue analogues generally leads to the formation of mixed materials with different structures and different compositions (due to weak variations in the synthesis procedure, to the presence of impurities) and, as a consequence, the synthesized materials are multiphasic materials that contribute to the coexistence of complex and different binding mechanisms.

An alternative process has been reported by Milyutin *et al.* [8] for the recovery of ^137^Cs^+^ by co-precipitation of the target alkali metal ion with successive additions of potassium ferrocyanide and metal nitrate (at molar ratio Me^2+^/Fe(CN)_6_ close to 1.33). Mixed precipitates of transition metal ferrocyanides and hydroxides are suggested to contribute to the reactive entrapment of radionuclide. The reaction is very sensitive to pH, due to the intrinsic stability of the precipitates and nickel ferrocyanide appears to be the most stable and the most efficient for Cs^+^ removal in alkaline solutions.

Nilchi *et al.* [136] also reported the possibility to prepare binary potassium-metal hexacyanoferrate by contact of the pre-formed potassium-nickel hexacyanoferrate with a copper solution: copper is partially exchanged with nickel to form the binary potassium-copper-nickel hexacyanoferrate.

The oxidation state of iron in the ion-exchanger was also suspected to strongly impact sorption properties [25,29]. Iron(III)-form has a stable structure that is not affected by Cs(I) sorption (with low sorption capacity and slow uptake). On the other hand, iron(II)-form may readily transform its structure for the insertion of larger quantities of Cs(I) [29]: the reaction appears to be kinetically controlled by ion-pair diffusion [25]. In addition, the presence of alkali metal ions (such as K(I)) strongly improves Cs(I) sorption capacity due to the supplementary contribution of ion-exchange mechanisms [29].

### 2.5. Performance and Process Limitations: The Rationale for Ion-Exchanger Immobilization

The ion-exchanger stability may depend on the pH, and the structure of the composite (mixed metal hexacyanoferrate). For example Milyutin *et al.* [75] commented that a partial dissolution of the hexacyanoferrate compounds occurs at pH above 11. Loos-Neskovic *et al.* [74] also reported the stability of potassium-copper hexacyanoferrate in neutral solution while, even in absence of cesium, the initial structure of the ion-exchanger is destroyed. The pH may contribute to the release of counter metals, which, in turn, affects the structure of the ion-exchanger (and its affinity or capacity to exchange metal ions, or make accessible site vacancies). The chemical and the physical stabilities are important criteria since the change in the structure of the ion-exchanger or the change in the crystalline structure may induce diffusion limitations, metal leakage and radionuclide release. This is especially important for both sorption performance and long-term storage of metal-loaded materials.

Depending on the structure of the ion-exchanger, its composition and the sorption mechanisms involved in metal binding the zeolite structure is generally favorable to diffusion of small ions and fast kinetics; however, as pointed by Loos-Neskovic *et al.* [17], in some cases, the crystal structure may change during metal sorption involving much slower kinetics. The small size of ion-exchanger particles (which depends on the mode of synthesis, as already reported) is a very important advantage for these materials since it allows reaching very high specific surface area, and then achieving very short equilibrium times. In the synthesis of copper ferricyanide (Cu_2_Fe(CN)_6_·7H_2_O) Han *et al.* [70] obtained a main fraction of particles close to 11.2 µm (with a Gaussian distribution), while a small fraction of the ion-exchanger had a size centered around 0.55 µm. Moon *et al.* [137] synthesized potassium cobalt hexacyanoferrate for the recovery of Cs and obtained particles in the range 2–19 µm (with an average value close to 8.5 µm). Ismail *et al.* [39] investigated the sorption of cobalt with potassium nickel hexacyanoferrate complex and demonstrated that (a) sorption capacity decreases with the increase of the size of ion-exchanger particles; and (b) that the uptake kinetics are enhanced when reducing the size of the material. Decreasing the size of Prussian Blues particles (ferric hexacyanoferrate, Fe^III^_4_[Fe^II^(CN_6_)_3_]) also improved the sorption of cesium [26], and thallium [95]. Similar trends were reported by Lehto and Harjula [101] for cesium recovery using potassium cobalt hexacyanoferrate and potassium copper cobalt hexacyanoferrate.

However the small size of these particles and their colloidal state may represent also a serious drawback for large-scale applications since the free decantation, the filtration of this micron-size materials make the solid/liquid separation quite complex. For example, in the case of co-precipitation of cesium with nickel ferrocyanide Milyutin and Gelis [7] reported the necessity to use a filtering material with a pore size no more than 0.2 µm. This means the necessity of frequent management of sophisticated equipment for the regeneration of the filtration medium and for maintaining appropriate filtration flow rate. A complementary treatment may be necessary to facilitate the recovery of spent ion-exchanger (metal-loaded sorbent): coagulation-flocculation has been suggested as a potential complementary treatment in order to agglomerate these nano- or microparticles [138]. Sinha *et al.* [139] recommend the use of ferric ions or, in some cases, polyelectrolytes, such as polyacrylamide (depending on the composition of the solution, especially the ionic strength, and the risk to promote metal exchange of ferric ions with the metal on the ion exchanger and the release of Cs^+^) for enhancing the recovery of ion-exchanger particles.

The stability issue and the difficulty in achieving a fast and efficient solid/liquid separation may explain the interest in managing the size of ion-exchanger particles and justify the numerous studies performed, for the last decades, for: (a) granulating these ion-exchangers; (b) processing their immobilization at the surface or in the porosity of appropriate supports; or (c) their encapsulation in a suitable matrix.

## 3. Techniques for Immobilization of Metal Hexacyanoferrates

The immobilization of metal hexacyanoferrate in/on supports may proceed through different processes depending on the type of support and the procedure for the synthesis of the ion-exchanger [1]. The process can make profit of (a) the encapsulating properties of the support (in this case the ion-exchanger should be pre-synthesized); or (b) the affinity of the support for one of the precursors (for example binding of the metal followed by the reaction of sodium or potassium hexacyanoferrate for *in situ* synthesis).

The mode of utilization of the material strongly influences the shaping of the ion-exchanger. As already reported the bulk material (nanometric or micrometric size) can be used directly in the wastewater by *in situ* synthesis and co-precipitation, or in the presence of pre-formed powder. The main drawback consists in the difficulty to readily recover the used material at the end of the processing. Frequently coagulation-flocculation is required to facilitate to solid/liquid separation [139]. Alternatively, centrifugation can be used but at the expense of more complex processes for large scale applications [135]. Combining metal hexacyanoferrate reactive groups with a magnetic core may also contribute to facilitate the solid/liquid separation [44,60,140,141]. The rationale of the immobilization is linked to the confinement of the active material in a much opened structured support (large specific surface area, appropriate size of pores). The shapings the most frequently reported are the granular form [10,59,76,78,104,142] or the manufacturing of beads [18,22,23,36,57,61,94,123,143,144]; however, some more innovative shapes have been also designed such as films or membranes [67,109,145,146,147] (mainly for electrochemical processes for cesium recovery or for sensor applications), foams or sponges [148,149,150,151,152,153].

Obviously, granular or powder-form of ion-exchangers are essentially applicable in batch systems with appropriate coagulation-flocculation (playing with the neutralization of charges of the ion-exchanger at the end of the sorption step) using Fe^3+^ or polyelectrolytes (anionic, neutral, cationic based on natural by-products or synthetic polyacrylamide and co-polymers) [139] or by centrifugation.

**Figure 3:**
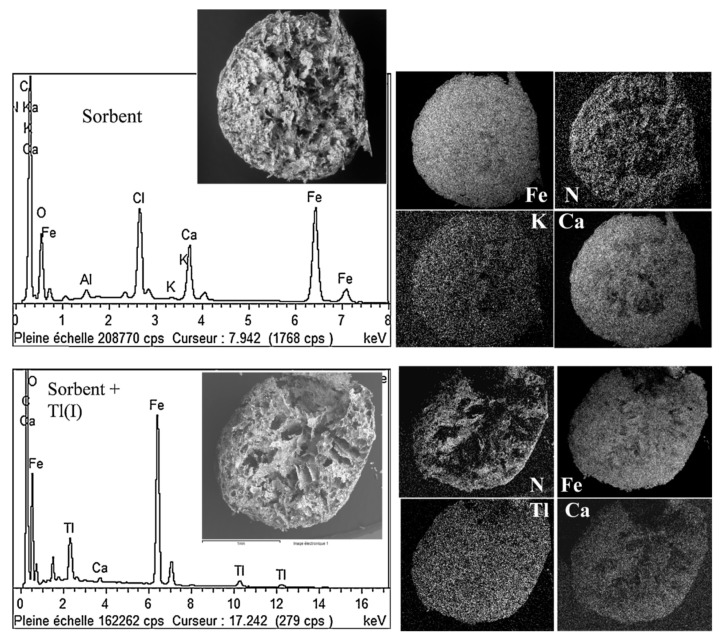
SEM-EDX analyses of cross-sections of hybrid Prussian Blue/alginate capsules before and after Tl(I) sorption (reprinted with permission from Elsevier [164]).

When conditioned under the form of beads, the typical mode of application is the fixed-bed column. These materials are similar to conventional ion-exchange or chelating resins. A series of columns can be used with the objective of saturating the sorbent and preventing the loss of target metal. In the case of conventional metal ions the desorption step is important for recovering the metal and for recycling/re-using the sorbent; however, in the case of radioelement, desorption step is useless and the critical parameter is the safe management of radioactive loaded material. In this case the column is considered a single-use cartridge that must be readily sealed for appropriate storage. Obviously, the parameters the most important for the design of these materials are the size of the beads (for reducing diffusion limitations), the size of the pores (for both limiting the resistance to intraparticle diffusion and the appropriate confinement of the nano- and micro-particles), the mechanical resistance (attrition, tensile and compressive strength). Mechanical properties of the material can be improved adding reinforcing fibers; for example in the case of ion-exchanger/chitin hybrid materials cellulose fibers have been incorporated in the synthesis and the mechanical properties have been improved [152]. The homogeneous distribution of the ion-exchanger into the support, and the effective accessibility of all the reactive sites are important for the optimization of the process. Figure 3 shows an example of SEM-EDX analysis of Prussian Blue/alginate capsules tested for the recovery of Tl(I). The element distribution of Fe and K element (as tracers of the ion-exchanger) in the capsule shows that Prussian Blue is homogeneously distributed in the whole mass of the sorbent (calcium element, as the tracer of the encapsulating material; *i.e.*, alginate, is also homogeneously distributed in the cross section). After metal sorption, thallium is also distributed in the whole mass of the sorbent.

### 3.1. Immobilization on the Support Matrix

#### 3.1.1. Inorganic Support

Mimura *et al.* [52] impregnated chabazite (a natural zeolite) with nickel nitrate (under reduced pressure to enhance the diffusion of nickel in the porous network of the zeolite) before reacting the impregnated material (again under reduced pressure) with K_4_[Fe(CN)_6_]. SEM analysis showed the precipitation of potassium-nickel hexacyanoferrate spherical/cubic particles at the surface (including internal porosity) of the zeolite. The same procedure (repeated for several successive impregnation steps) was used for the synthesis of potassium-nickel hexacyanoferrate/SiO_2_ sorbent [53]. As expected the distribution coefficients and the sorption capacities increased with the number of impregnation cycles (due to the increase of metal hexacyanoferrate immobilized on the support): the maximum sorption capacity increased almost linearly (from 0.14 to 0.3 mmol Cs·g^−1^) with increasing the ion-exchanger loading from 11% to 24% (*w*/*w*). The size of the precipitates of metal hexacyanoferrate was in the range 20–50 nm; this is an important criteria since for silica gel of large specific surface area (but low average pore diameter) the crystals are too large and metal hexacyanoferrate is mainly located at the external surface of the support: this means that supports with the smallest size of pores may have limited capacity to support metal hexacyanoferrate and may, in turn, lead to lower sorption capacities because of the progressive blocking of pore channels during the successive impregnation/synthesis steps. It is noteworthy that the crystal lattice of metal hexacyanoferrate on silica gel support was very close to the values obtained with the conventional precipitation process (*i.e.*, 1.01 ± 0.01 for both precipitate and encapsulated forms [19,52,53]). Kazemian *et al.* [43] applied a very similar procedure for the immobilization of potassium-nickel hexacyanoferrate in the porosity of clinoptilolite and other zeolite material (obtained by hydrothermal alkaline treatment of clinoptilolite): successive impregnation batches (under reduced pressure) were used for the *in situ* synthesis of the ion exchanger. They investigated the effect of temperature on the distribution coefficient of Cs^+^ and Sr^2+^: while strontium removal was poorly affected by the temperature the distribution coefficient for cesium progressively increased with temperature.

After synthesizing MCM-41 mesoporous silica, Vo *et al.* [105] immobilized potassium-cobalt hexacyanoferrate (K_0.84_Co_1.08_[Fe(CN)_6_]) on the porous support using successive batches of cobalt nitrate in methanol/water solutions and K_3_[Fe(CN)_6_] aqueous solutions: the order of impregnation is reversed compared to most of the papers reporting successive impregnation procedures. The size of synthesized nanocrystals was evaluated by XRD analysis (through the Scherrer equation) to approximately 2 nm, consistently with the pore size of MCM-41: the authors comment that the channels of the mesoporous material act as nanoscale reactor for the synthesis of metal hexacyanoferrate particles. The issue of pore blockage reported by Mimura *et al.* [19,52,53] was not documented (probably because of the lower amount of ion-exchanger loaded on the support); however, the TEM images showed that the porous structure of the MCM-41 silica support was not affected by immobilization of PB analogue; though the specific surface of impregnated material was much higher (between 260 and 438 m^2^·g^−1^, depending on the type of ion-exchanger) than the value obtained with as-produced MCM-41 (*i.e.*, 7 m^2^·g^−1^). Milyutin *et al.* [51] used a series of mineral supports (natural calcium carbonate, aluminosilicates (bentonite, clinoptilolite) and silica-based materials (diatomite, biosilica)) for *in situ* synthesis of potassium-nickel hexacyanoferrate sorbents: the mineral supports are first impregnated with nickel sulfate before being mixed with an excess of potassium hexacyanoferrate. The distribution coefficients for Cs were systematically higher than those obtained under similar experimental conditions with bulk potassium-nickel hexacyanoferrate.

Folch *et al.* [154] proposed an alternative method for the incorporation of metal hexacyanoferrate: after preparing hybrid silica (SBA-15 type, bearing NCH_5_H_4_(CH_2_)_2_ functionalities), they impregnated the support with metal chloride species in methanol; finally the dried material was mixed with a methanol solution containing [N(C_4_H_9_)_4_]_3_[Fe(CN)_6_] (but other cyano-metallate salts can be also used, based, for example, on Mo or Co). The sequence of impregnation steps was repeated to produce different layers of metal hexacyanoferrate. The size of crystal metal hexacyanoferrate was directly controlled by the pore size of the silica matrix used as the hosting material. Delchet *et al.* [99] used almost the same procedure for the deposition of metal hexacyanoferrate on silica matrices and porous glasses: metal impregnation was performed (in successive cycles) in methanol solution using Co(BF_4_) as the impregnating salt and reacting with tetrabutylammonium hexacyanoferrate. Very small (10 nm size-order) crystals of cobalt hexacyanoferrate were immobilized in the porosity of the supports.

Magnetic Prussian Blue particles were prepared by synthesis of magnetic nanoparticles (by co-precipitation of ferrous and ferric salts (Fe(III)/Fe(II) molar ratio: 2/1) with ammonium hydroxide. After washing with water and ethanol, the dried black precipitate was reacted with K_4_[Fe(CN)_6_] (improving the dispersion of magnetic nanoparticles under ultrasonication), after adjusting the pH to 2, a blue precipitate of PB/magnetic particles was obtained [155].

Sangvanich *et al.* [79] functionalized MCM-41 silica with ethylenediamine terminated silane ([3-(2-aminoethyl-3-amino)propyl]trimethoxysilane, deposited on silica in refluxing toluene through a series of self-assembled monolayers procedure, [156]) for further binding copper ions (from CuCl_2_ aqueous solution). Prior to contact the resulting copper-silica composite with a sodium hexacyanoferrate solution, the solid was treated in toluene under reflux with a Dean-Stark trap (to condensate all free Si-OH groups). Compared to Prussian Blue insoluble ion-exchanger the supported potassium-copper hexacyanoferrate showed much higher sorption capacities for Cs^+^ and improved selectivity.

Liu *et al.* [143] immobilized titanium onto silica-based supports by contact with Ti(OC_4_H_9_)_4_ in cyclohexane (under heating at 80 °C and phase condenser, and under nitrogen atmosphere), prior to reaction with K_4_[Fe(CN)_6_] solution (in 0.5 M HCl): Ti atoms were not fully substituted on the support and TiO_2_ sites remained on the support; the general formula of the potassium-titanium hexacyanoferrate was close to K_2x_[(TiO_2_)_2−x_Fe(CN)_6_] (with x close to 0.5). The high specific surface area of the supports was only slightly decreased after hexacyanoferrate immobilization (other textural parameters such as pore volume and average pore size were also hardly affected). Very high distribution coefficients were obtained: the presence of sodium salts (i.e., nitrate or chloride) halved K_d_ values (when passing from 0.1 M to 1 M) but this effect was less drastic than the effect of HNO_3_ concentration (decrease by two orders of magnitude when increasing acid concentration from 0.1 M to 1 M, while HCl concentration did not influence K_d_).

Voronina’s Group investigated the functionalization of TiO_2_ with metal hexacyanoferrate [62,124,125,157]. Basically, the support (initially under the form of hydrated TiO_2_) was converted into its hydrogen-sodium form (by successive treatment with HCl and NaOH solutions to pH 6–7), followed by the successive steps: (a) calcination of the support at 400 °C (increase in mechanical stability); (b) impregnation with nickel sulfate, and, finally; (c) the reaction of bound nickel with K_4_[Fe(CN)_6_] to form the potassium-nickel hexacyanoferrate at the surface of the titanium oxide support. They reported the interest of the mixed titanium dioxide/potassium-nickel hexacyanoferrate composite for the sorption of both Sr^2+^ (on titanium dioxide) and Cs^+^ (on metal hexacyanoferrate).

The grafting of propyl-ethylenediamine triacetate on silica allows binding Ni(II), which, in turn, acts as an anchoring site for the deposition of nickel hexacyanoferrate layers [34]. In this case, nickel ions are sorbed on chemically-modified silica particles, before the metal-loaded particles are immersed in a K_4_[Fe(CN)_6_] solution. The resulting material has a good affinity for Cs^+^ even in the presence of large excess of K^+^: distribution coefficient approaches 2 × 10^6^ mL·g^−1^.

Quartz and indium-tin oxide (ITO) modified glasses were coated with different Prussian Blue analogues [90]. The supports were pre-treated with poly(diallyldimethylammonium chloride) or with poly(allylamine chloride). The pre-treated substrates were immersed in successive baths containing aqueous solutions of (a) potassium hexacyanoferrate (either HCF(II) and HCF(III), depending on the type of metal hexacyanoferrate to be synthesized) and (b) metal chloride solutions (or ammonium ferrous sulfate). The synthesized films were used as separating membranes: the selectivity factor and the fluxes depended on the relative size of hydrated metal ions compared to the size of zeolite channels in Prussian Blue analogues.

Sharygin’s Group investigated the sorption properties of Termosksid-35 a zirconium hydroxide support coated with K_2_Ni[Fe(CN)_6_] (ion-exchanger content reaches up to 32%–36%, *w*/*w*) [59,158,159]: they reported the strong stability of the composite materials at storage in terms of sorption properties and structure.

#### 3.1.2. Polymer

Ion-exchange resins have frequently been used for immobilizing hexacyanoferrate-based ion-exchangers into their porous network by local precipitation using a sequence of successive contact steps with solutions of the two precursors (potassium/sodium hexacyanoferrate and metal salt) [58,111]. The order in the sequence of contacts is not fixed and the literature shows many examples of both hexacyanoferrate/metal salt and metal salt/hexacyanoferrate sequences. IRA-904 (a macroreticular anion exchange resin holding quaternary amine functional groups) was first converted to its hydroxide form for binding potassium ferrocyanide [81]. The copper hexacyanoferrate resin was finally obtained by contact with a copper nitrate solution. The sorbent was very selective for cesium recovery (compared to other alkali metal ions). A similar procedure was used for synthesizing iron hexacyanoferrate resin: after binding potassium ferrocyanide the resin was mixed with ferric nitrate [126]. This resin was very efficient for the chromatographic separation of radionuclides playing with the type and concentration of eluent. The same concept was applied by the same research group for preparing metal hexacyanoferrate IRA-904 resins (using either copper, iron(III) or nickel chloride ions) and the sorbents were very efficient for the recovery and analysis of ^137^Cs^+^ and ^125^I^−^ and ^125^IO_3_^−^ in water and milk [64]. Similar procedure was used for depositing cobalt ferrocyanide into the porous network of a strong base anion exchange resin (polystyrene divinyl benzene polymer backbone bearing –N(CH_3_)_3_^+^ groups) [104,122]: the anion-exchanger resin was mixed with potassium hexacyanoferrate before adding cobalt nitrate; the process is supposed precipitating Co_2_Fe(CN)_6_ inside resin network. A post-treatment with a KCl solutions allowed partially exchanging cobalt with potassium to form potassium-cobalt hexacyanoferrate into the resin [122]: the ion-exchanger structure was found close to K_1.28_Co_1.36_[Fe(CN)_6_]. Won *et al.* [65] used IRN-78 strong base anion exchange resin for impregnation/reaction with potassium ferrocyanide followed by contact with copper nitrate, cobalt nitrate or nickel nitrate to synthesize potassium-(copper, or cobalt or nickel) hexacyanoferrate.

Clarke and Wai [68] used a Chelex-20 resin for binding copper nitrate before reacting Cu-resin with K_4_[Fe(CN)_6_] solution. The decrease in Cs sorption properties that was observed when the composite resin was in contact with nitric acid solutions was attributed to an oxidative phenomenon that converted Fe^II^(CN)_6_^4−^ into Fe^III^(CN)_6_^3−^. Adding hydrazine or ascorbic acid into the solution contributed to reduce the oxidation mechanism and to maintain the sorption efficiency of the composite material.

Macroporous anion exchange resins (pre-conditioned in the chloride form) were equilibrated twice with a potassium hexacyanoferrate solution before the impregnated resins were reacted with a mixture of copper sulfate and potassium sulfate [58].

Non-woven polypropylene PP fabrics were chemically modified by grafting carboxylic groups (though a radiation-induced graft polymerization of acrylic acid monomer on the fabric) [32]. The carboxylate-bearing PP was loaded with nickel ions by adsorption from a NiCl_2_ solution. The Ni-loaded support was immersed into a K_4_[Fe(CN)_6_] solution to form the potassium-nickel hexacyanoferrate/PP composite. Yasutaka *et al.* [96] and Tsuji *et al.* [93] reported the use of non-woven fabrics impregnated with Prussian Blue for the analysis and recovery of cesium. However, the process used for the impregnation procedure was not fully described. The PB-impregnated discs were stacked inside cylindrical columns for radio-cesium uptake.

#### 3.1.3. Biopolymer

Kitajima *et al.* [87] impregnated cotton gauze with water-dispersible Prussian Blue particles (characterized by the structure: Fe_4_[Fe(CN)_6_]·Na_4.2_[Fe(CN)_6_]_1.05_·15H_2_O, with particle size ranging between 10 and 20 nm); sodium cations were then exchanged with K^+^ to make the metal hexacyanoferrate insoluble and stabilized on cotton gauze. Heating the composite at 135 °C allowed reinforcing the stability of PB nanoparticles. Very high decontamination factors were obtained, and the SEM-EDX analysis clearly showed that K^+^ ions were exchanged with Cs^+^ after contact with the radionuclide solution.

Pre-formed chitosan gel beads have been prepared by Rumyantseva *et al.* [78] and they have been used for copper sorption (from copper sulfate or copper nitrate aqueous solutions) before being mixed with K_4_[Fe(CN)_6_] solutions (two-fold excess compared to the amount of copper bound to chitosan). XRD analysis showed that the metal hexacyanoferrate immobilized on the gel beads was the phase K_2_Cu_3_[Fe(CN)_6_]_2_. The materials produced from copper nitrate salts were characterized by lower distribution coefficient than those produced from sulfate salts. Mass transfer appeared to be controlled by the resistance to external diffusion.

Another technique for the *in situ* deposition of metal ferrocyanide in alginate matrices has been described by Tokarev *et al.* [61]: first, the alginate solution was ionotropically gelled by reaction of carboxylate functions with metal cations (from Ni(II), Cu(II), Fe(II), Mn(II) and Eu(III)). In a second step, water was exchanged with acetonitrile. Metal/alginate material was then mixed with an acetonitrile solution containing [N(C_4_H_9_)_4_]_3_[M’(CN)_m_] (with M’: Fe^3+^, Cr^3+^ (m = 6) or Mo^5+^ (m = 8)). This intermediary product was then reacted with an acetonitrile solution containing the metal nitrate salt used for the ionotropic gelation to coordinate these ions onto the cyanometallate core. The sequence of operations can be repeated in order to increase the size of metal hexacyanoferrate crystals. These materials were characterized for their photo-luminescent properties (with Eu^3+^ complex) or magnetic properties (other metal complexes).

#### 3.1.4. Carbon-Based Support

Activated carbon and carbon nanotubes have been successfully used for the immobilization of metal hexacyanoferrate complexes. These materials are well known for their high porosity and large specific surface area that enhance the contact between the ion-exchanger and target metal ions. They can be used either as direct support (direct immobilization of metal hexacyanoferrates) or as functionalized support (large specific surface materials supporting a polymer that will entrap the metal hexacyanoferrate complexes). The immobilization of the ion-exchanger (K_2_Cu[Fe(CN)_6_]) on activated carbon contributes to reduce the pore volume of the support and the specific surface area of the raw material (from 528 to 161 m^2^·g^−1^) [80]. The synthesis of the composite material consisted in a two-step procedure: first activated carbon was impregnated with copper sulfate (under vacuum) followed by the contact (under agitation) with a potassium hexacyanoferrate (K_4_[Fe(CN)_6_]) solution (molar ratio Cu/K: 2). SEM-EDX analysis showed the precipitation of the hexacyanoferrate complex at both the internal and external surfaces of the support. A similar procedure was used for the immobilization of potassium iron(III) hexacyanoferrate on activated carbon by impregnation of the support with K_4_[Fe(CN)_6_], drying before contacting with FeCl_3_·6H_2_O (followed by washing and drying) [86]. The ion-exchanger (of structure close to K_3x_ Fe_4-x_ [Fe(CN)_6_]_3_) was essentially localized in the macropores of activated carbon (*i.e.*, pores of 400-4000 nm). The sorbent showed great efficiency for Cs removal from sea water.

Sheveleva *et al.* [117,118] reported the deposition of a Co,Ni-hexacyanoferrate/siloxane-acrylate emulsion at the surface of activated carbon fibers. The siloxane-acrylate emulsion was mixed, for several successive steps, with cobalt and nickel chloride solutions and with potassium hexacyanoferrate. The stable metal hexacyanoferrate emulsion (negative surface charge) was deposited at the surface of positively-charged carbon fibers by electrodeposition in 0.1 M NaCl solutions (potential varying between +0.3 and +0.9 V, without polarization). The thickness of the film and the size of aggregates deposited at the surface of the fibers increase with the potential applied to the system. Increasing the concentration of the emulsion also contributes to (a) its coagulation under electrodeposition conditions; and (b) to produce flaky films, which, in turn, lead to a decrease in the distribution coefficient for Cs removal. Jeerage *et al.* [160] also reported the cathodic deposition of nickel hexacyanoferrate on a suitable electrode for reverse electrochemical sorption/desorption of cesium.

Hong *et al.* [83] synthesized a three dimensionally ordered macroporous (3DOM) carbon matrix for the immobilization of Prussian Blue (PB) nanoparticles. The 3DOM carbon matrix was then mixed with FeCl_3_ and K_3_[Fe(CN)_6_] solution under ultrasonic irradiation. The composite materials have been successfully tested for the recovery of ^133^Cs, ^85^Rb, ^138^Ba, ^88^Sr, ^140^Ce and ^205^Tl (for target application in radionuclide decorporation: elimination in the intestinal and esophagus tract). The affinity of the material increases with ionization energy of the target metal (from ^205^Tl to ^133^Cs). Under the same experimental conditions the sorption capacities of immobilized PB for target radionuclides were about 300 times greater than those obtained with bulk PB, and about 30 times those of Radiogardase^®^.

More recently, Yang *et al.* [66] used multiwalled carbon nanotubes (MWCNT) as a support for the immobilization of nickel hexacyanoferrate through poly(4-vinylpyridine) grafting (P4VP). First the MWCNT was mixed (under ultrasonic dispersion conditions) with 4-ethylenepyridine (under nitrogen atmosphere) before adding K_2_S_2_O_8_ as the initiator of the polymerization. Nickel nitrate was mixed with K_3_[Fe(CN)_6_] in acetic acid solution in the presence of P4VP-MWCNT: the metal hexacyanoferrate is formed and grows in the framework of the polymer coating while MWCNTs offer large specific surface area for improved efficiency of nickel hexacyanoferrate. They reported the use of these composite materials as supercapacitors.

#### 3.1.5. Miscellaneous

Biosorbents have been also used for the immobilization of metal hexacyanoferrates. For example, Jalali-Rad *et al.* [40] used algal biomass pre-treated with EDTA for binding copper ions. In a second step potassium hexacyanoferrate (K_3_[Fe(CN)_6_]) was contacted with Cu-EDTA-algae material for the *in-situ* precipitation of potassium-copper hexacyanoferrate complex. Similar Cs sorbents were prepared replacing copper ions with nickel ions to form potassium-nickel hexacyanoferrate complex. The procedure drastically increases the affinity of the different algal materials for Cs. A similar process was used for the impregnation of potassium-nickel hexacyanoferrate into a lignocellulosic material (*i.e.*, coir pith) [56]. The support was mixed with nickel chloride solutions prior contacting the Ni-Coir pith with a potassium hexacyanoferrate solution. The entire procedure was repeated for a total of 5 cycles of impregnation/reaction in order to increase potassium-nickel hexacyanoferrate loading. The incorporation of metal hexacyanoferrate doubled Cs sorption capacity of the material. Vrtoch *et al.* [63] used the Jalali-Rad procedure for incorporating potassium nickel hexacyanoferrate complex in the caps of macro fungi *Agaricus bisporus* (*i.e.*, successive contacts of the fungi biomass with nickel nitrate solution and potassium hexacyanoferrate solutions). Walnut shells (pre-treated with HCl) have been functionalized by successive impregnation with NiCl_2_ and K_3_[Fe(CN)_6_] solutions [6,161].

### 3.2. Encapsulation-Entrapment

#### 3.2.1. Sol-Gel

A simple sol-gel procedure was described by Orechovska and Rajec [55] for the immobilization of potassium-nickel hexacyanoferrate (and sodium-nickel hexacyanoferrate). Sodium silicate solution was mixed with potassium (or sodium) hexacyanoferrate before adding an acidic solution of nickel nitrate. The ion-exchangers precipitated in the silica gel formed during neutralization. In order to prepare spherical beads, the methods were slightly modified [57]. The reaction took place in an oil phase: the aqueous phase containing the reagents was dispersed under strong agitation in the oil phase, the silica gel is supposed to form simultaneously (or little later) than the potassium-nickel hexacyanoferrate ion exchanger. Liu *et al.* [103] synthesized potassium-cobalt hexacyanoferrate that was immobilized in SiO_2_ by a sol-gel procedure (using silicate sodium for building the entrapment matrix): very high metal hexacyanoferrate loadings were obtained (close to 70% *w*/*w*).

Ali *et al.* [82] used rice straw for the extraction of silica-based material that was, in a second step, converted to a zeolite (zeolite synthesis method) by hydrothermal treatment (mixing silica with aluminate, sodium hydroxide oxide, *n*-propyl amine and tetrapropylammonium bromide, followed by reaction at pH 11 with a solution of aluminum sulfate in H_2_SO_4_, under heating at 140 °C). After calcination of the precipitate (obtained at pH 8) at 550 °C, the support was impregnated with K_3_[Fe(CN)_6_] and dried. An alternative sorbent was produced introducing directly the K_3_[Fe(CN)_6_] or K_4_[Fe(CN)_6_] salt in the sol-gel solution: this method is supposed to build-up the zeolite crystals around the complexes (the complexes are taking the place of aluminum element in the zeolite channels).

Causse *et al.* [162] synthesized potassium-copper hexacyanoferrate by fast reaction of copper nitrate with K_4_[Fe(CN)_6_] and they introduced the ion-exchanger in a mixture of tetraethoxysilane (TEOS) and acidic solution of Pluronic P-123 (triblock copolymer) as the precursors of silica monolith (dodecane was added as a surfactant for stabilizing the emulsion). Sodium fluoride was added for initiating the polycondensation of silica and after one week of curing the composite potassium-copper hexacyanoferrate/silica monolith was obtained. The monoliths were characterized by very high BET surface area (ranging between 278 and 643 m^2^·g^−1^, depending on the percentage of ion-exchanger nanoparticles in the composite).

#### 3.2.2. Polymer

The coagulation of polyacrylonitrile (PAN) in water solution can be used to form fibers, beads, membranes, granular particles. For example, PAN dissolved in dimethylsulfoxide (DMSO) can be coagulated into a coagulation bath of DMSO/water [163]; beads were formed by drops [36], fiber by spinning, and membrane by casting. Incorporating the precursors of the ion-exchanger (*i.e.*, nickel nitrate and K_4_[Fe(CN)_6_]) in the PAN solution allows its encapsulation after coagulation [136]. The materials have been successfully tested for ^137^Cs and ^60^Co recovery from radioactive wastes: the sorbent has a marked preference for cesium over cobalt. Copper hexacyanoferrate (obtained by reaction of potassium hexacyanoferrate with copper nitrate) was mixed with PAN in DMSO and the mixture was dropped in water for preparing copper hexacyanoferrate/PAN composite sorbent [77]. The addition of tween-80 allows improving the quality, dispersion and sphericity of composite beads. The BET surface area of sorbent particles exceeded 75 m^2^·g^−1^. Distribution coefficient increased with pH while it slightly decreased in the presence of alkali and alkaline-earth metal ions. PAN was also used by Kamenik *et al.* [114] for incorporating potassium-nickel hexacyanoferrate and for the successful recovery of ^134^Cs and ^137^Cs from seawater. Sheha [60,131] used PAN for the entrapment of magnetic nano/microparticles that were functionalized by potassium-zinc hexacyanoferrate complex [60] or potassium-nickel hexacyanoferrate complex [131].

The same concept was used for the synthesis of copper hexacyanoferrate/polymer composite beads using polyethersulfone (PES) (in the presence of *N*,*N*-methyl pyrrolidone, MP) as the encapsulating material [22]: copper sulfate was added to a potassium hexacyanoferrate solution in the presence of PVA (for reducing the size of copper hexacyanoferrate particles). The dried precipitate was incorporated in the PES/MP solution prior to be distributed in a water bath.

Potassium-copper hexacyanoferrate and potassium-nickel hexacyanoferrate (previously prepared by reaction of precursors: copper or nickel chloride solutions with K_4_[Fe(CN)_6_]) were immobilized together with magnetite by polycondensation of sulfonated phenol and formaldehyde [73]. The analysis of the ion-exchangers shows the coexistence of different hexacyanoferrate forms (differences in composition and structure). The sorbents were tested for ^212^Pb sorption and the distribution coefficient decreases with either nitric or sulfuric acid concentrations. Metal desorption exceeds 90% when using 1 M HCl solutions.

Polyurethane (PU) was used as an encapsulating material for immobilizing a composite made of Prussian Blue and diatomite that was *in situ* produced by impregnation of diatomite with iron(III) chloride solution and further reaction with Na_4_[Fe(CN)_6_] [84]. The immobilization of PB in the porosity network of the silica-based material was reinforced with multi-walled carbon nanotubes (MWCNTs) prior to be incorporated in PU (zwitterionic surfactants were added as wetting agent, while sulfonated-surfactants were added as dispersant). The PU foam (PUF) containing the dried composite material was obtained by mixing the composite with polyurethane pre-polymer (NB-90000B, derived from poly(oxy C2-4 alkylene)diol and toluene diisocyanate) before reacting with water. The encapsulated composite material was very stable and efficient for cesium sorption (including ^137^Cs). A similar procedure was used by Tsuruoka *et al.* [121] for cesium removal: high distribution coefficients were obtained in sea water and fresh water; the salt content of sea water hardly changed K_d_.

#### 3.2.3. Biopolymer

**Figure 4:**
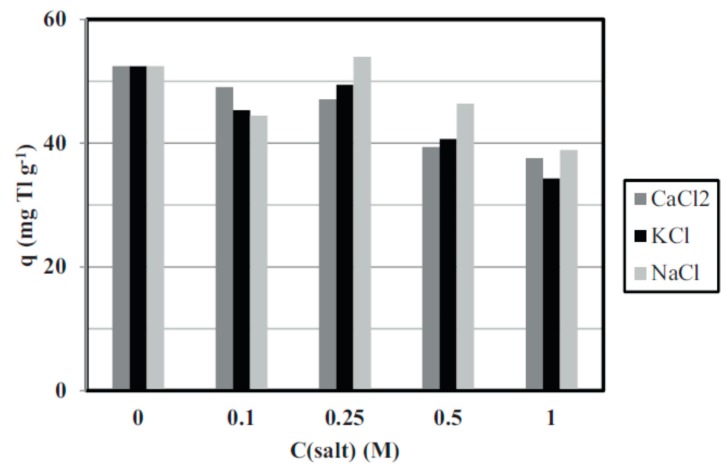
Effect of increasing concentration of salts (of alkali and alkaline-earth metal salts) on Tl(I) sorption capacity (reprinted with permission from Elsevier, [164]).

Contrary to the previous cases cited for the *in situ* synthesis of metal hexacyanoferrate on pre-formed alginate beads, in the present method the gel bead formation was simultaneous or posterior to the synthesis of the ion-exchanger. For example, potassium-copper hexacyanoferrate was immobilized by a sol-gel method in alginate gel polymers and the sorbent was used for Pd^2+^ recovery from high level liquid wastes [130]. The ion-exchanger particles were homogeneously distributed in the whole mass of the alginate beads. In complex solutions the analysis of distribution of metal showed the selectivity of the ion-exchanger for Pd ions while other significant metals (such as Ru and Zr) were bound to the encapsulating matrix. Alginate was used by Vipin *et al.* for the encapsulation of Prussian Blue (either soluble or insoluble forms; *i.e.*, Fe_4_[Fe(CN)_6_]_3_ and KFe_3_[Fe(CN)_6_]_3_, respectively) [94], and sodium-cobalt hexacyanoferrate [123], directly or through carbon nanotube (CNTs) immobilization. The colloidal Prussian Blue solution was mixed with CNTs suspension and then added to alginate solution. The viscous complex solution was then distributed dropwise into a calcium chloride solution for ionotropic gelation (between carboxylate functions and calcium ions). XRD and FTIR analyses confirm the presence of PB. SEM analysis shows the presence of porous channels with a concentric distribution (poorly interconnected): this structure is poorly affected by the presence of CNTs [94]. Similar trends were reported in the case of the encapsulation of sodium-cobalt hexacyanoferrate with alginate (and CNTs) [123]. Prussian Blue was synthesized by adding drop by drop K_4_[Fe(CN)_6_] solution into a FeCl_3_ solution. The washed precipitate was dried and further incorporated into a sodium alginate solution before being ionotropically gelled by dropping the mixture into a CaCl_2_ solution [164]. The composite material was successfully used for thallium recovery. SEM-EDX analysis showed that the ion-exchanger was homogeneously dispersed into the composite material and that Tl element was also present in the whole volume of the bead; this means that all reactive sites remain available in the material. Tested in the presence of Rb(I) the sorption of thallium was hardly affected by the competitor ion: the sorbent is very specific to Tl(I) [164]: the presence of large excess of competitor cations (Na^+^, K^+^, Ca^2+^) hardly influenced sorption capacity (Figure 4).

Dwiwedi *et al.* [23] also applied the encapsulation procedure for the immobilization of potassium-cobalt hexacyanoferrate with alginate gel matrix. The precursors of the ion-exchanger (*i.e.*, CoSO_4_ and potassium hexacyanoferrate) were mixed in the presence of polyvinyl alcohol (which acts as a stabilizer of metal hexacyanoferrate complex and which contributes to control the size of nanoparticles). Sodium alginate solution was added to the suspension and the mixture was ionotropically gelled (dropwise addition in a calcium chloride solution).

**Figure 5:**
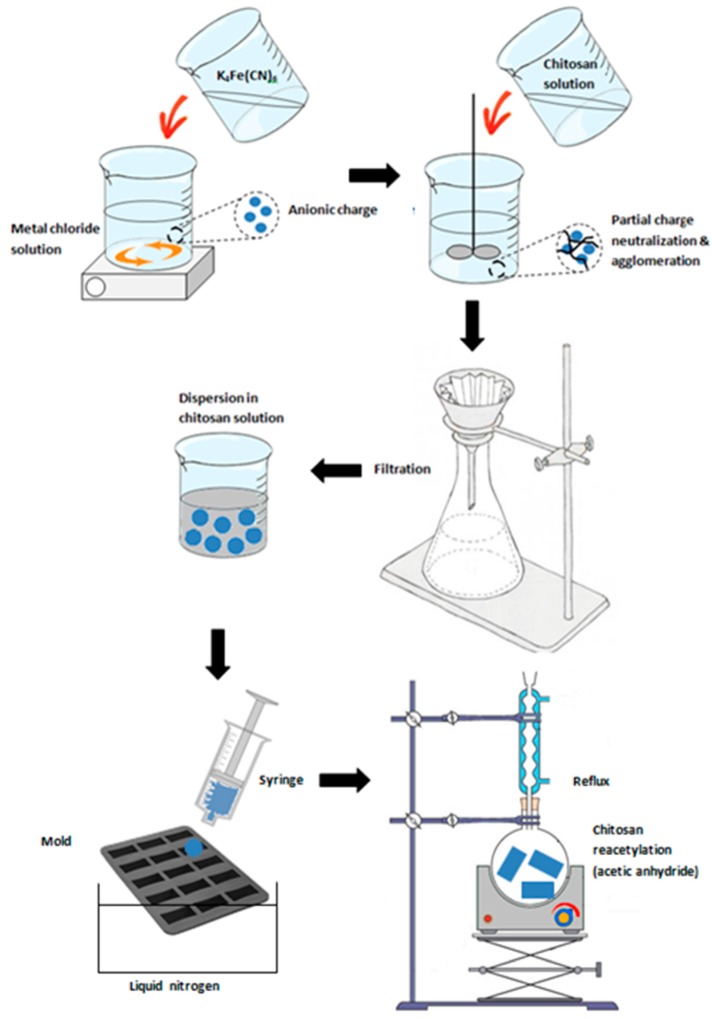
Schematic route for the synthesis of chitin sponge composites.

Recently, Prussian Blue analogues were incorporated into highly macroporous membranes or foams/sponges [18,153] made of chitin; Figure 5 schematically describes the synthesis route. Pre-synthesized potassium-nickel hexacyanoferrate (obtained by reaction of potassium hexacyanoferrate and nickel sulfate) was incorporated in chitosan solution (the biopolymer being dissolved in acetic acid solution) [152]. The suspension (homogeneously dispersed using an Ultra-Turrax) was poured in a Petri dish before being frozen at −80 °C. The frozen disc was then freeze-dried. The frozen water is forming a porous network in the frozen disc, water sublimation during the freeze-drying step maintains a macro-porous network. The freeze-drying process generates the high macroporosity of the composite (the macroporosity depends on the concentration of the chitosan solution, the freezing temperature, *etc*.). This high macroporosity contributes to the high permeation properties of these materials that can be disposed in column systems (fixed-bed columns). However, in order to minimize the preferential channels that could form it may be important to force high flow rates in the system and to recycle the solution (to achieve sufficient contact times) [152]: a minimum flow velocity is necessary for limiting these wall and dead volume effects.

**Figure 6:**
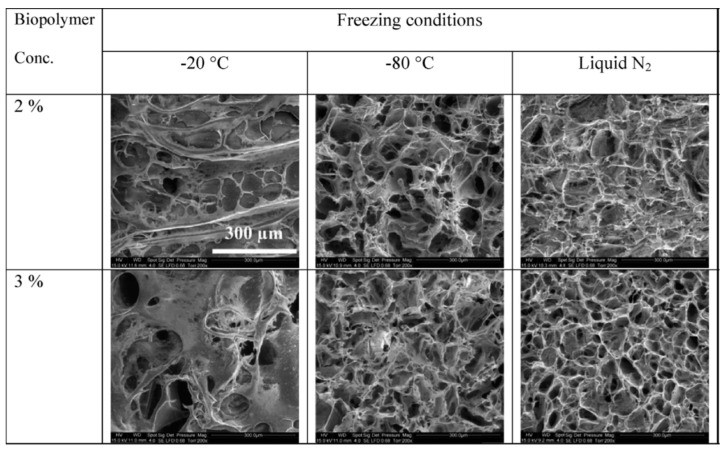
Textural properties of chitin sponges: effect of chitosan concentration and freezing temperature (reprinted with permission from Elsevier [153].

The ion-exchanger/chitosan foam may be unstable in acidic solutions (with the remarkable exception of sulfuric acid solutions), it is thus necessary reinforcing its stability in acidic solutions. This objective was reached by acetylation of amine groups (using acetic anhydride reaction with amine functions). The composite potassium-nickel hexacyanoferrate/chitin foam can be used for Cs(I) binding using the foams as reactive filtrating discs. Very efficient and promising breakthrough curves were obtained using a fixed-bed column filled with these discs. The porous properties of the materials are controlled by experimental parameters such as the concentration of chitosan solution and the freezing temperature. Figure 6 shows the influence of these parameters on the textural properties of the hybrid materials [153]. PB/chitin foams prepared through the same procedure were designed for specific applications for the recovery of accidental dumping of metal-bearing solutions [153]. The dried foams (conditioned as large thin sponges) can be used for absorbing contaminated discharged water; after a few minutes of contact/reaction, the treated water is drilled from the sponge (by gravity, centrifugation, or wringing, *etc.*) while the metal or the radionuclide is transferred from the dispersed water phase to the solid phase.

A similar concept was used by Vincent *et al.* [18] for manufacturing chitin beads: (a) different Prussian Blue analogues were synthesized using potassium hexacyanoferrate and a series of metal salts: Cu-, Ni-, Zn-, Co and Fe salts); (b) the potassium-metal hexacyanoferrates were incorporated into chitosan solution before being distributed drop by drop into a liquid N_2_ batch (for shape-forming); (c) the freeze-drying of the beads was followed by the acetylation of PB analogue/chitosan beads.

**Figure 7:**
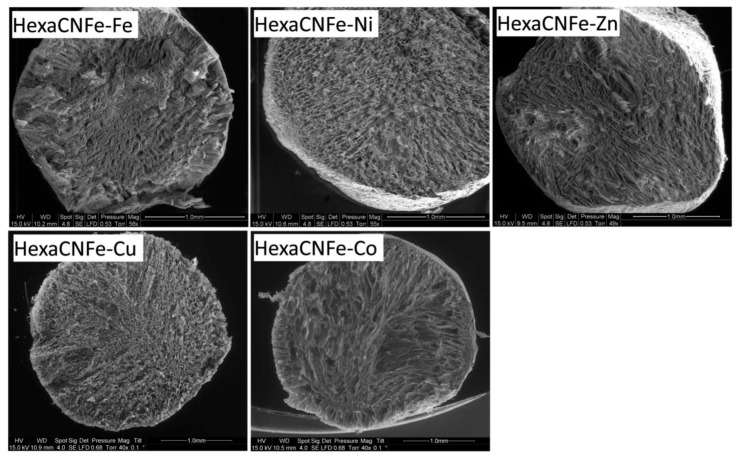
SEM analysis of cross-section of hybrid metal hexacyanoferrate/chitin beads (reprinted with permission from Royal Society of Chemistry [18]).

TEM-analysis of potassium-metal hexacyanoferrate showed very different structures between Zn-based material (large cubic particles) and other PB analogues (smaller and roughly spherical particles) (Figure 2). The freezing of the particles in liquid N_2_ contributes to forming a very porous structure. Again SEM-EDX analysis confirmed the homogeneous distribution and the great accessibility of reactive sites to Cs(I) ions. Figure 7 shows the porous structure of hybrid materials prepared by encapsulation of pre-synthesized Prussian Blue analogues in chitin beads formed by freezing in liquid N_2_ and further reacetylation [18].

The drying of the material (when required) may be critical since the irreversible drying can strongly impact the porosity of the material and its diffusion properties (accessibility, availability): in the case of hydrogels obtained by ionotropic gelation of biopolymer (alginate). Tokarev *et al.* [61] reported the use of drying under supercritical CO_2_ conditions for maintain the textural properties of the original material. They also described the synthesis of photo-luminescent films made of *in situ* produced cyano-bridged coordination polymer nanoparticles (based on Eu^3+^/[Mo(CN)8]^3−^): the *in situ* synthesis of sequential synthesis of the ion-exchanger composite film was already reported (See Section 3.1.3).

Basically the alginate solution was ionotropically gelled with a metal salt (alginate solution casted on a chromatography paper impregnated with the relevant metal salt solution) before exchanging water with acetonitrile. In a second step, the films were put in contact alternatively (and repeatedly) with acetonitrile solutions of [N(C_4_H_9_)_4_]_3_[M(CN)_m_] (with M = Mo, m = 8 for molybdenum-based cyanometallate) and metal nitrate salt (here [Eu^3+^(H_2_O)_6_](NO_3_)_3_). Similar films have been prepared with nickel and copper (instead of europium) using hexacyanoferrate (instead of cyanomolybdate complex) for manufacturing magnetic hybrid membranes. Films, membranes could be used as spiral modules for the reactive filtration of contaminated effluents.

### 3.3. Miscellaneous–Granulation and Magnetic Particles

The granulation of the material is directly associated to the drying procedure. While the freeze-drying partially contributes to manage capillary forces during the drying step and results in highly-dispersed particles, air-drying leads to agglomeration of particles that can be grinded at the appropriate size for practical application [165]. Increasing the size of ion-exchanger facilitates handling and management of the sorbent at the expense of a reduction in the availability and accessibility of reactive groups (which, in turn, affects the uptake kinetics and, in some cases, the equilibrium performance). Attrition phenomena may occur and Nilchi *et al.* [165] suggested impregnating the dried material with a 4% aqueous polyvinyl alcohol solution to improve the mechanical stability of the ion-exchanger.

Loos-Neskovic *et al.* [166] have developed a method they called “growth from the solid” that consists in the slow growth of the insoluble compound on a soluble reacting crystal placed in a concentrated solution of the other reactant. Basically, the alkaline hexacyanoferrate M^I^_4_[Fe(CN)_6_] crystal is introduced in a solution of a metal salt whose hexacyanoferrate complex is insoluble. A layer of this insoluble complex is formed at the surface of the alkaline hexacyanoferrate crystal. The progressive addition of new quantities of metal salt leads to the deposition of new layers of the insoluble complex. The method allows the preparation of granulated homogeneous spherical particles that can be practically used in fixed-bed systems.

Alternatively, the management of nano- and micrometric size sorbents can be facilitated using magnetic particles as support for the immobilization of metal hexacyanoferrates [44,131,141]. Kolodynska *et al.* [44] reported the synthesis of insoluble potassium-nickel hexacyanoferrates (by reaction of NiCl_2_ with K_4_Fe(CN)_6_), followed by the polycondensation of sulfonated phenol and formaldehyde in the presence of a mixture of the ion-exchanger with magnetite powder. Sheha *et al.* [131] proposed mixing anionic hexacyanoferrate species (added as K_4_Fe(CN)_6_) with positively charged magnetite nanoparticles (protonated ferrite particles). In a second step, nickel chloride was added to the mixture for precipitating potassium-nickel hexacyanoferrates as a thin layer at the surface of magnetite nanoparticles. The entrapment of these composite materials in polyacrylonitrile (PAN) gel did not improve Cu sorption properties and slightly slowed the uptake kinetics but maintained the confinement of micro-particles. More recently, Zhang *et al.* [141] immobilized potassium titanium hexacyanoferrate on 100-nm magnetite particles for the recovery of cesium. Magnetite particles were successively coated with SiO_2_ (by reaction of tetraethyl orthosilicate with sodium silicate in an ethanol/water solution) and TiO_2_ (by reaction of tetrabutyl titanate with isopropyl alcohol/water solution, followed by reaction with ammonia). The support was then reacted with K_4_[Fe(CN)_6_] to form a composite (Fe_3_O_4_/SiO_2_/K_4-y_Ti_x_[Fe(CN)_6_]) that was efficiently tested for cesium sorption.

Avramenko *et al.* [30,31] reported the synthesis of latex colloid particles made of siloxane-acrylate emulsions (particle size close to 160 nm) mixed with metal (cobalt, copper or nickel) chloride salts (single-metal salt or combination of metal salts) with potassium hexacyanoferrate at pH 7. The composite emulsion is relatively stable (for at least 1 month); however, at high hexacyanoferrate content non-stabilized particles are formed and can be recovered by settling or filtration [31]. Comparing the potential of these materials for flocculation of radionuclides with the performances obtained by cesium precipitation in presence of the precursors (direct co-precipitation process) they point out that the most significant advantage is the remarkable reduction in the volume of the sludge produced by the treatment (*i.e.*, 1% *vs.* 25%) [30]. They also reported the electrodeposition of cobalt hexacyanoferrate/latex emulsion at the surface of carbon fibers (with high specific surface area) [31].

The thermal treatment of a mixture containing polyvinylpyrrolidone (PVP) and K_3_[Fe(CN)_6_] (in HCl solution) leads to the crystallization of Prussian Blue particles [167]. Actually, Hu *et al.* showed that the potassium hexacyanoferrate(III) is partially decomposed to release iron(III) ions that are reduced by PVP. Iron(II) reacts with potassium hexacyanoferrate(III) to form Fe_4_[Fe(CN)_6_]. PVP plays the role of the reductant and contributes to the control of the size and the shape of PB particles.

Polymethylmethacrylate (PMMA) was used for preparing structured granular ion-exchanger [91,120]: PMMA was suspended in water (in the presence of a surfactant); K_4_[Fe(CN)_6_] and ferrous sulfate solutions were successively added. After pH control to 0.69 with sulfuric acid a green precipitate was obtained. After careful washing the precipitate was oxidized with successive batches of potassium chlorate and hydrochloric acid solutions. The granulated potassium iron(III)hexacyanoferrate supported PMMA material was dried, grinded and sieved.

An original process was described by Chen *et al.* [67,109] for the electro-adsorption of cesium using copper hexacyanoferrate deposited (by spin coating) on gold electrodes. Copper(II) hexacyanoferrate(III) was first synthesized (as a mixture of Cu_3_[Fe(CN)_6_]_2_ and KCu[Fe(CN)_6_] using copper nitrate and K_3_[Fe(CN)_6_] as precursors; the hydrophilicity of the product was increased by mixing with Na_4_[Fe(CN)_6_] solutions. The copper hexacyanoferrate “ink” was spin coated on the working electrode (reference and counter electrodes were Hg/HgCl_2_/KCl (saturated solution) and platinum electrodes, respectively). The main advantages of the process are: (a) the absence of consumption of chemical reagents and of sludge production; (b) the reversibility of the reaction (potential switching between anodes and cathodes); and (c) easy and fast sorbent separation from treated solution.

For health application (*i.e.*, metal decorporation) it may be useful to immobilize the ion-exchanger in a suitable matrix, compatible with physiological conditions and stable in the digestive tract to reach the appropriate location for optimized absorption in the body. It means that it remains stable in either acidic or alkaline conditions to follow the digestive tract and deliver adequate amounts of Prussian Blue. Indeed, administration of bulk Prussian Blue requires absorption of excessive amounts that could be modulated with a suitable support. Biopolymers with controlled pH range of stability appear to be promising materials for this kind of application.

### 3.4. Stability of Structured Materials

The challenges for the structured materials consist in the stability of composite materials. This stability issue deals with mechanical stability (effective confinement of nano- or micro-particles in the hybrid materials) [22], chemical stability (acid range, presence of salts) [125,140], radiation stability (especially important for polymer and biopolymers encapsulating materials submitted to radiolysis conditions) [33,136].

The confinement of active materials (*i.e.*, the micro- or nano-size ion-exchanger particles) is a key parameter since it controls the stability of the ion-exchanger and controls the solid/liquid separation; this is even more critical in the case of radionuclide entrapment: the confinement of metal-loaded ion-exchanger particles in the matrix is an important requisite. If the process of immobilization consists in the physical entrapment the matrix porosity should not be affected by swelling effect. For example, in the case of encapsulation in alginate beads [164] the ionotropic gelation with calcium chloride can be reversed in alkaline solution: calcium cation being exchanged with sodium ion the biopolymer capsule partially dissolves and ion-exchanger particles may be released. Similar phenomena can be observed with chitosan-based materials: in acidic solutions (except in sulfuric acid media) chitosan can dissolve and release the ion-exchanger; the problem was solved by chemical modification of the biopolymer: the reacetylation of chitosan into chitin (conversion of glucosamine into *N*-acetyl-d-glucosamine) improves the chemical stability of the biopolymer in acidic solutions [18,153]. Nilchi *et al.* [136] also reported weak chemical stability of polyacrylonitrile matrices for the encapsulation of potassium nickel hexacyanoferrate: the polymer partially or totally dissolves in concentrated acids, or in some specific solutions (such as ZrCl_2_, LiBr, CaCl_2_ or NaSCN). The stability of magnetic supports in acidified solutions is also of critical importance: usually below pH 2, magnetite begins to hydrolyze and partially dissolve.

The resistance to radiolysis is also an important criterion for the design of immobilized ion-exchangers. Inorganic supports are generally more stable to irradiation effects than polymer-based materials [168,169], though in some cases the gamma irradiation may also impact: (a) the structure of the ion-exchanger, which, in turn, influences its topographical properties (cage effect) and its affinity for target metal [21,169]; (b) the chemical composition of the ion-exchanger (oxidation of Fe(II) to Fe(III), for example, with possible effect on the reactivity of the ion-exchanger) [33]. In the case of gamma irradiation (at the dose of 4 MGy) of potassium nickel ferrocyanide deposited on silica gel support, Bykov *et al.* [33] showed that the ^137^Cs distribution coefficient decreased by a factor of 2.5; However, the sorbent maintained a quite high distribution coefficient (>10^4^) that confirms the relatively high stability of the composite. Irradiation may impact the molecular weight of the polymer [168] (with a potential effect on the tensile properties of the material), the mechanical properties of the composite [170], pore size characteristics [171], their intrinsic ion-exchange properties [172], *etc.* The gamma irradiation may cause chemical decomposition of organic material producing hydrogen with potential explosive hazard. Preliminary tests performed on chitin-based encapsulation of Prussian Blue analogues have shown a relative stability and no production of hydrogen gas at low irradiation (unpublished results). This criterion of stability is important in the processing of metal sorption, but this is also a critical issue for the long-term stability of radionuclide-loaded ion-exchanger: the radiolysis of the loaded composite may increase the mobility of the radioelement and limits the potential of the material for long-term storage.

Note: In a recent paper Olatunji *et al.* [173] review the sorption of cesium using a series of sorbents including hexacyanoferrate-based materials. They comment on the effect of experimental parameters (specific sorption parameters) and discuss the relevant stability issues. The reader can consult this review oriented toward sorption performance, which is complementary to the present mini-review on synthesis of composite materials.

## 4. Conclusions

A great number of processes have been designed for the last decades for the immobilization of ion-exchangers based on potassium-metal hexacyanoferrates. These hybrid materials are very efficient for the recovery of radionuclides and analogues (such as radioelement issued from cesium, rubidium and thallium). The hybrid materials can be synthesized by incorporation and entrapment (or encapsulation) of pre-formed ion-exchanger micro- or nano-particles in a suitable matrix (polymer or biopolymer, or sol-gel process for mineral supports) or by direct *in situ* synthesis of the ion-exchanger micro- or nano-particles into the porous network of pre-formed supports.

Critical parameters are the confinement of the micro- and nano-particles, the size of pores of the supports (and subsequent diffusion properties), the stability of the composites (in terms of both mechanical, chemical and radiolysis properties), the density (or proportion) of ion-exchanger particles in the hybrid material.

The objective of the wastewater treatment and the mode of use of the hybrid material conditions the selection of the process used for the synthesis of the ion-exchanger composite: encapsulation usually performed with polymer-based supports offer a great diversity of conditioning but at the expense of a limited resistance against radiolysis. Mineral supports offer good mechanical and radiolysis resistance; however, the loading of ion-exchanger is generally limited in percentage and except a few examples of monolithic mineral supports the choice in shaping is rather limited.

Apart from these applications of supported hybrid materials in environmental remediation, these metal-hexacyanoferrate composites can be used for other applications such as gas capture and separation [42,174], as sensors [145,147], in energy applications [66], in synthesis of photo-luminescent materials [175,176]. De Taconi *et al.* [28] reviewed different applications of metal hexacyanoferrates (such as magneto-optic/optic-magneto switching, energy conversion, display devices and smart windows, photo-imaging, *etc.*) that could probably find applications when supported on a suitable matrix.

## Supplementary Materials

Supplementary materials can be accessed at: http://www.mdpi.com/1420-3049/20/11/19718/s1.

## References

[1] Gaur S. (1996). Determination of Cs-137 in environmental water by ion-exchange chromatography. J. Chromatogr. A.

[2] Thompson D.F., Callen E.D. (2004). Soluble or insoluble Prussian blue for radiocesium and thallium poisoning?. Ann. Pharmacother..

[3] Thompson D.F., Church C.O. (2001). Prussian blue for treatment of radiocesium poisoning. Pharmacotherapy.

[4] Melo D.R., Lipsztein J.L., Leggett R., Bertelli L., Guilmette R. (2014). Efficacy of Prussian Blue on Cs-137 decorporation therapy. Health Phys..

[5] Heyltex Radiogardase. http://www.heyltex.com/products/radiogardase.

[6] Ding D., Zhao Y., Yang S., Shi W., Zhang Z., Lei Z., Yang Y. (2013). Adsorption of cesium from aqueous solution using agricultural residue-Walnut shell: Equilibrium, kinetic and thermodynamic modeling studies. Water Res..

[7] Milyutin V., Gelis V. (2008). Optimal conditions for coprecipitation of cesium radionuclides with nickel ferrocyanide. Radiochemistry.

[8] Milyutin V., Mikheev S., Gelis V., Kononenko O. (2009). Coprecipitation of microamounts of cesium with precipitates of transition metal ferrocyanides in alkaline solutions. Radiochemistry.

[9] Lehto J., Haukka S., Harjula R., Blomberg M. (1990). Mechanism of cesium ion-exchange on potassium cobalt hexacyanoferrates(II). J. Chem. Soc., Dalton Trans..

[10] Haas P.A. (1993). A review of information on ferrocyanide solids for removal of cesium from solutions. Sep. Sci. Technol..

[11] Ayrault S., Loos-Neskovic C., Fedoroff M., Garnier E. (1994). Copper hexacyanoferrates—Preparation, composition and structure. Talanta.

[12] Ismail I., El-Sourougy M., Moneim N., Aly H. (1998). Preparation, characterization, and utilization of potassium nickel hexacyanoferrate for the separation of cesium and cobalt from contaminated waste water. J. Radioanal. Nucl. Chem..

[13] Ayrault S., Loos-Neskovic C., Fedoroff M., Garnier E., Jones D.J. (1995). Compositions and structures of copper hexacyanoferrates(II) and (III): Experimental results. Talanta.

[14] Loos-Neskovic C., Fedoroff M., Garnier E. (1989). Preparation, composition and structure of some nickel and zinc ferrocyanides: Experimental results. Talanta.

[15] Loos-Neskovic C., Fedoroff M., Garnier E., Gravereau P. (1984). Zinc and nickel ferrocyanides—Preparation, composition and structure. Talanta.

[16] Lee E.F.T., Streat M. (1983). Sorption of cesium by complex hexacyanoferrates. 5. A comparison of some cyanoferrates. J. Chem. Technol. Biotechnol. A.

[17] Loos-Neskovic C., Fedoroff M. (1989). Fixation mechanisms of cesium on nickel and zinc ferrocyanides. Solvent Extr. Ion Exch..

[18] Vincent T., Vincent C., Barre Y., Guari Y., Le Saout G., Guibal E. (2014). Immobilization of metal hexacyanoferrates in chitin beads for cesium sorption: Synthesis and characterization. J. Mater. Chem. A.

[19] Mimura H., Kageyama N., Akiba K., Yoneya M., Miyamoto Y. (1998). Ion-exchange properties of potassium nickel hexacyanoferrate(II) compounds. Solvent Extr. Ion Exch..

[20] Ishfaq M.M., Karim H.M.A., Khan M.A. (1992). Preparation and characterization of potassium copper nickel hexacyanoferrate(II) as an ion exchanger for cesium. J. Radioanal. Nucl. Chem..

[21] Gaffar M.A., Omar M.H. (2005). Thermal analytical study of different phases of potassium hexacyanoferrate(II) crystal—Effects of growth conditions, heat treatment and gamma-irradiation on the unit cell parameters. J. Therm. Anal. Calorim..

[22] Dwivedi C., Kumar A., Singh K.K., Juby A.K., Kumar M., Wattal P.K., Bajaj P.N. (2013). Copper hexacyanoferrate-polymer composite beads for cesium ion removal: Synthesis, characterization, sorption, and kinetic studies. J. Appl. Polym. Sci..

[23] Dwivedi C., Pathak S.K., Kumar M., Tripathi S.C., Bajaj P.N. (2013). Potassium cobalthexacyanoferrate-gel beads for cesium removal: Kinetics and sorption studies. RSC Adv..

[24] Rodriguez-Hernandez J., Reguera E., Lima E., Balmaseda J., Martinez-Garcia R., Yee-Madeira H. (2007). An atypical coordination in hexacyanometallates: Structure and properties of hexagonal zinc phases. J. Phys. Chem. Solids.

[25] Ayrault S., Jimenez B., Garnier E., Fedoroff M., Jones D.J., Loos-Neskovic C. (1998). Sorption mechanisms of cesium on Cu^II^_2_Fe^II^(CN)_6_ and Cu^II^_3_[Fe^III^(CN)_6_]_2_: Hexacyanoferrates and their relation to the crystalline structure. J. Solid State Chem..

[26] Faustino P.J., Yang Y., Progar J.J., Brownell C.R., Sadrieh N., May J.C., Leutzinger E., Place D.A., Duffy E.P., Houn F. (2008). Quantitative determination of cesium binding to ferric hexacyanoferrate: Prussian blue. J. Pharm. Biomed. Anal..

[27] Le Gall B., Taran F., Renault D., Wilk J.C., Ansoborlo E. (2006). Comparison of Prussian blue and apple-pectin efficacy on ^137^Cs decorporation in rats. Biochimie.

[28] De Tacconi N.R., Rajeshwar K., Lezna R.O. (2003). Metal hexacyanoferrates: Electrosynthesis, *in situ* characterization, and applications. Chem. Mater..

[29] Grandjean A., Delchet C., Causse J., Barré Y., Guari Y., Larionova J. (2015). Effect of the chemical nature of different transition metal ferrocyanides to entrap Cs. J. Radioanal. Nucl. Chem..

[30] Avramenko V., Bratskaya S., Zheleznov V., Sheveleva I., Marinin D., Sergienko V. (2011). Latex particles functionalized with transition metals ferrocyanides for cesium uptake and decontamination of solid bulk materials. Proceedings of the 13th International Conference on Environmental Remediation and Radioactive Waste Management, ICEM2010.

[31] Avramenko V., Bratskaya S., Zheleznov V., Sheveleva I., Voitenko O., Sergienko V. (2011). Colloid stable sorbents for cesium removal: Preparation and application of latex particles functionalized with transition metals ferrocyanides. J. Hazard. Mater..

[32] Bondar Y., Kuzenko S., Han D.H., Cho H.K. (2014). Development of novel nanocomposite adsorbent based on potassium nickel hexacyanoferrate-loaded polypropylene fabric. Nanoscale Res. Lett..

[33] Bykov G., Milyutin V., Ershov B., Korchagin Y., Gelis V., Bessonov A. (2011). Radiation resistance of a composite ferrocyanide-silica gel sorbent. Radiochemistry.

[34] Chang C.Y., Chau L.K., Hu W.P., Wang C.Y., Liao J.H. (2008). Nickel hexacyanoferrate multilayers on functionalized mesoporous silica supports for selective sorption and sensing of cesium. Microporous Mesoporous Mater..

[35] Chen Y., Wang J. (2012). Removal of radionuclide Sr^2+^ ions from aqueous solution using synthesized magnetic chitosan beads. Nucl. Eng. Des..

[36] Du Z., Jia M., Wang X. (2013). Cesium removal from solution using PAN-based potassium nickel hexacyanoferrate(II) composite spheres. J. Radioanal. Nucl. Chem..

[37] Galamboš M., Suchánek P., Rosskopfová O. (2012). Sorption of anthropogenic radionuclides on natural and synthetic inorganic sorbents. J. Radioanal. Nucl. Chem..

[38] Ishfaq M.M., Karim H.M.A., Khan M.A. (1997). A radiochemical study on the thermodynamics of cesium adsorption on potassium copper nickel hexacyanoferrate(II) from aqueous solutions. J. Radioanal. Nucl. Chem..

[39] Ismail I.M., El-Sourougy M.R., El-Moneim N.A., Aly H.F. (2002). The sorption of cobalt from aqueous solutions by potassium nickel hexacyanoferrate complex. Solvent Extr. Ion Exch..

[40] Jalali-Rad R., Ghafourian H., Asef Y., Dalir S.T., Sahafipour M.H., Gharanjik B.M. (2004). Biosorption of cesium by native and chemically modified biomass of marine algae: introduce the new biosorbents for biotechnology applications. J. Hazard. Mater..

[41] Jeerage K.M., Steen W.A., Schwartz D.T. (2002). Charge-density-dependent partitioning of Cs^+^ and K^+^ into nickel hexacyanoferrate matrixes. Langmuir.

[42] Karadas F., El-Faki H., Deniz E., Yavuz C.T., Aparicio S., Atilhan M. (2012). CO_2_ adsorption studies on Prussian blue analogues. Microporous Mesoporous Mater..

[43] Kazemian H., Zakeri H., Rabbani M.S. (2006). Cs and Sr removal from solution using potassium nickel hexacyanoferrate impregnated zeolites. J. Radioanal. Nucl. Chem..

[44] Kołodyńska D., Hubicki Z., Kubica B. (2012). Hexacyanoferrate composite sorbent in removal of anionic species from waters and waste waters. Sep. Sci. Technol..

[45] Kopyrin A.A., Pyartman A.K., Keskinov V.A., Pleshkov M.A., Sobolev I.A., Dmitriev S.A. (1999). Cesium-selective composites. I. Synthesis and ion-exchange properties of sorbents based on AV-17 anion exchanger and double ferrocyanides of transition metals and potassium. Radiochemistry.

[46] Kopyrin A.A., Pyartman A.K., Keskinov V.A., Pleshkov M.A., Sobolev I.A., Dmitriev S.A. (1999). Cesium-selective composites. II. Synthesis and ion-exchange properties of sorbents based on VP-1Ap anion exchanger and double ferrocyanides of transition metals and potassium. Radiochemistry.

[47] Kopyrin A.A., Pyartman A.K., Keskinov V.A., Pleshkov M.A., Sobolev I.A., Dmitriev S.A. (2000). Cesium-selective composites: III. Synthesis and ion-exchange properties of sorbents based on AMP anion exchanger and double ferrocyanides of transition metals and potassium. Radiochemistry.

[48] Kubica B., Tuteja-Krysa M., Misiak R., My T.T.T., Kubica M., Stobinski M., Godunowa H. (2003). The behavior of Ba and Sr on inorganic and organic ion-exchangers from sulphuric acid solutions—Preliminary experiments. J. Radioanal. Nucl. Chem..

[49] Loos-Neskovic C., Dierkes M.H., Jackwerth E., Fedoroff M., Garnier E. (1993). FIixation of palladium on insoluble simple or complex cyano compounds. Hydrometallurgy.

[50] Loos-Neskovic C., Pedoroff M. (1987). Exchange mechanisms of silver on nickel and zinc ferrocyanides. Solvent Extr. Ion Exch..

[51] Milyutin V., Kononenko O., Mikheev S., Gelis V. (2010). Sorption of cesium on finely dispersed composite ferrocyanide sorbents. Radiochemistry.

[52] Mimura H., Kimura M., Akiba K., Onodera Y. (1999). Selective removal of cesium from sodium nitrate solutions by potassium nickel hexacyanoferrate-loaded chabazites. Sep. Sci. Technol..

[53] Mimura H., Kimura M., Akiba K., Onodera Y. (1999). Selective removal of cesium from highly concentrated sodium nitrate neutral solutions by potassium nickel hexacyanoferrate(II)-loaded silica gels. Solvent Extr. Ion Exch..

[54] Mostafa M., El-Absy M.A., Amin M., El-Amir M.A., Farag A.B. (2011). Partial purification of neutron-activation (99)Mo from cross-contaminant radionuclides onto potassium nickel hexacyanoferrate(II) column. J. Radioanal. Nucl. Chem..

[55] Orechovska J., Rajec P. (1999). Sorption of cesium on composite sorbents based on nickel ferrocyanide. J. Radioanal. Nucl. Chem..

[56] Parab H., Sudersanan M. (2010). Engineering a lignocellulosic biosorbent—Coir pith for removal of cesium from aqueous solutions: Equilibrium and kinetic studies. Water Res..

[57] Rajec P., Orechovska J., Novak I. (2000). NIFSIL: A new composite sorbent for cesium. J. Radioanal. Nucl. Chem..

[58] Ramaswamy M. (1997). Sorption of cesium by hexacyanoferrate composites from neutral and acidic media. Solvent Extr. Ion Exch..

[59] Sharygin L., Muromskiy A., Kalyagina M., Borovkov S. (2007). A granular inorganic cation-exchanger selective to cesium. J. Nucl. Sci. Technol..

[60] Sheha R.R. (2012). Synthesis and characterization of magnetic hexacyanoferrate(II) polymeric nanocomposite for separation of cesium from radioactive waste solutions. J. Colloid Interface Sci..

[61] Tokarev A., Agulhon P., Long J., Quignard F., Robitzer M., Ferreira R.A.S., Carlos L.D., Larionova J., Guerin C., Guari Y. (2012). Synthesis and study of Prussian blue type nanoparticles in an alginate matrix. J. Mater. Chem..

[62] Voronina A.V., Semenishchev V.S., Nogovitsyna E.V., Betenekov N.D. (2013). Peculiarities of sorption isotherm and sorption chemisms of caesium by mixed nickel-potassium ferrocyanide based on hydrated titanium dioxide. J. Radioanal. Nucl. Chem..

[63] Vrtoch Ľ., Pipíška M., Horník M., Augustín J., Lesný J. (2011). Sorption of cesium from water solutions on potassium nickel hexacyanoferrate-modified *Agaricus bisporus* mushroom biomass. J. Radioanal. Nucl. Chem..

[64] Watari K., Imai K., Ohmomo Y., Muramatsu Y., Nishimura Y., Izawa M., Baciles L.R. (1988). Simultaneous adsorption of Cs-137 and I-131 from water and milk on “metal ferrocyanide-anion exchange resin”. J. Nucl. Sci. Technol..

[65] Won H.J., Moon J.K., Jung C.H., Chung W.Y. (2008). Evaluation of ferrocyanide anion exchange resins regarding the uptake of Cs^+^ ions and their regeneration. Nucl. Eng. Technol..

[66] Yang Y., Hao Y., Wang X., Yan Q., Yuan J., Shao Y., Niu L., Huang S. (2015). Controllable synthesis of coaxial nickel hexacyanoferrate/carbon nanotube nanocables as advanced supercapcitors materials. Electrochim. Acta.

[67] Chen R., Tanaka H., Kawamoto T., Asai M., Fukushima C., Kurihara M., Watanabe M., Arisaka M., Nankawa T. (2012). Preparation of a film of copper hexacyanoferrate nanoparticles for electrochemical removal of cesium from radioactive wastewater. Electrochem. Commun..

[68] Clarke T.D., Wai C.M. (1998). Selective removal of cesium from acid solutions with immobilized copper ferrocyanide. Anal. Chem..

[69] Han F., Zhang G.-H., Gu P. (2012). Removal of cesium from simulated liquid waste with countercurrent two-stage adsorption followed by microfiltration. J. Hazard. Mater..

[70] Han F., Zhang G.-H., Gu P. (2013). Adsorption kinetics and equilibrium modeling of cesium on copper ferrocyanide. J. Radioanal. Nucl. Chem..

[71] Ishfaq M.M., Karim H.M.A., Khan M.A. (1993). Adsorption studies of cesium on potassium copper-nickel hexacyanoferrate(II) from aqueous solutions. J. Radioanal. Nucl. Chem..

[72] Jimenez-Gallegos J., Rodriguez-Hernandez J., Yee-Madeira H., Reguera E. (2010). Structure of porous copper prussian blue analogues: Nature of their high H-2 storage capacity. J Phys. Chem. C.

[73] Kubica B. (2010). Sorption of lead(II) on copper(II) and nickel-potassium hexacyanoferrates and magnetite-loaded resin from inorganic acid solutions. Nukleonika.

[74] Loos-Neskovic C., Ayrault S., Badillo V., Jimenez B., Garnier E., Fedoroff M., Jones D.J., Merinov B. (2004). Structure of copper-potassium hexacyanoferrate (II) and sorption mechanisms of cesium. J. Solid State Chem..

[75] Milyutin V., Mikheev S., Gelis V., Kozlitin E. (2009). Sorption of cesium on ferrocyanide sorbents from highly saline solutions. Radiochemistry.

[76] Nilchi A., Malek B., Maragheh M.G., Khanchi A. (2003). Investigation of the resistance of the potassium copper nickel hexacyanoferrate(II) ion exchanger against gamma irradiation. Radiat. Phys. Chem..

[77] Nilchi A., Saberi R., Moradi M., Azizpour H., Zarghami R. (2011). Adsorption of cesium on copper hexacyanoferrate-PAN composite ion exchanger from aqueous solution. Chem. Eng. J..

[78] Rumyantseva E., Veleshko A., Kulyukhin S., Veleshko I., Shaitura D., Rozanov K., Dmitrieva N. (2009). Preparation and properties of modified spherically granulated chitosan for sorption of ^137^Cs from solutions. Radiochemistry.

[79] Sangvanich T., Sukwarotwat V., Wiacek R.J., Grudzien R.M., Fryxell G.E., Addleman R.S., Timchalk C., Yantasee W. (2010). Selective capture of cesium and thallium from natural waters and simulated wastes with copper ferrocyanide functionalized mesoporous silica. J. Hazard. Mater..

[80] Wang L., Feng M., Liu C., Zhao Y., Li S., Wang H., Yan L., Tian G., Li S. (2009). Supporting of potassium copper hexacyanoferrate on porous activated carbon substrate for cesium separation. Sep. Sci. Technol..

[81] Watari K., Imai K., Izawa M. (1967). Isolation of ^137^Cs with copper ferrocyanide-anion exchange resin. J. Nucl. Sci. Technol..

[82] Ali I.O., Salama T.M., Thabet M.S., El-Nasser K.S., Hassan A.M. (2013). Encapsulation of ferro- and ferricyanide complexes inside ZSM-5 zeolite synthesized from rice straw: Implications for synthesis of Prussian blue pigment. Mater. Chem. Phys..

[83] Hong J.-Y., Oh W.-K., Shin K.-Y., Kwon O.S., Son S., Jang J. (2012). Spatially controlled carbon sponge for targeting internalized radioactive materials in human body. Biomaterials.

[84] Hu B., Fugetsu B., Yu H., Abe Y. (2012). Prussian blue caged in spongiform adsorbents using diatomite and carbon nanotubes for elimination of cesium. J. Hazard. Mater..

[85] Hu M., Furukawa S., Ohtani R., Sukegawa H., Nemoto Y., Reboul J., Kitagawa S., Yamauchi Y. (2012). Synthesis of prussian blue nanoparticles with a hollow interior by controlled chemical etching. Angew. Chem. Int. Ed..

[86] Kawatake K., Shigemoto N. (2012). Preparation of potassium iron(III) hexacyanoferrate(II) supported on activated carbon and Cs uptake performance of the adsorbent. J. Nucl. Sci. Technol..

[87] Kitajima A., Tanaka H., Minami N., Yoshino K., Kawamoto T. (2012). Efficient cesium adsorbent using Prussian Blue nanoparticles immobilized on cotton matrices. Chem. Lett..

[88] Liu H., Du X., Liang C., Liu P., Xu J., Fang J., Shen W., Zhao J. (2010). Morphologies and magnetic properties of cobalt-iron Prussian Blue analogues nanoparticles synthesized in microemulsion. Synth. React. Inorg. Met.-Org. Nano-Met. Chem..

[89] Namiki Y., Namiki T., Ishii Y., Koido S., Nagase Y., Tsubota A., Tada N., Kitamoto Y. (2012). Inorganic-organic magnetic nanocomposites for use in preventive medicine: A rapid and reliable elimination system for cesium. Pharm. Res..

[90] Pyrasch M., Toutianoush A., Jin W.Q., Schnepf J., Tieke B. (2003). Self-assembled films of Prussian blue and analogues: Optical and electrochemical properties and application as ion-sieving membranes. Chem. Mater..

[91] Taj S., Ashraf Chaudhry M., Mazhar M. (2009). Potassium iron(III)hexacyanoferrate(II) supported on polymethylmethacrylate ion-exchanger for removal of strontium(II). J. Radioanal. Nucl. Chem..

[92] Torad N.L., Hu M., Imura M., Naito M., Yamauchi Y. (2012). Large Cs adsorption capability of nanostructured Prussian Blue particles with high accessible surface areas. J. Mater. Chem..

[93] Tsuji H., Kondo Y., Suzuki Y., Yasutaka T. (2012). Development of a method for rapid and simultaneous monitoring of particulate and dissolved radiocesium in water with nonwoven fabric cartridge filters. J. Radioanal. Nucl. Chem..

[94] Vipin A.K., Hu B., Fugetsu B. (2013). Prussian blue caged in alginate/calcium beads as adsorbents for removal of cesium ions from contaminated water. J. Hazard. Mater..

[95] Yang Y., Faustino P.J., Progar J.J., Brownell C.R., Sadrieh N., May J.C., Leutzinger E., Place D.A., Duffy E.P., Yu L.X. (2008). Quantitative determination of thallium binding to ferric hexacyanoferrate: Prussian blue. Int. J. Pharm..

[96] Yasutaka T., Kawamoto T., Kawabe Y., Sato T., Sato M., Suzuki Y., Nakamura K., Komai T. (2013). Rapid measurement of radiocesium in water using a Prussian blue impregnated nonwoven fabric: Fukushima NPP Accident Related. J. Nucl. Sci. Technol..

[97] Terada K., Hayakawa H., Sawada K., Kiba T. (1970). Silica gel as a support for inorganic ion-exchangers for the determination of caesium-137 in natural waters. Talanta.

[98] Ca D.V., Cox J.A. (2004). Solid phase extraction of cesium from aqueous solution using sol-gel encapsulated cobalt hexacyanoferrate. Microchim. Acta.

[99] Delchet C., Tokarev A., Dumail X., Toquer G., Barre Y., Guari Y., Guerin C., Larionova J., Grandjean A. (2012). Extraction of radioactive cesium using innovative functionalized porous materials. RSC Adv..

[100] Hirayama Y., Okamura Y., Fujiwara K., Sugo T., Umeno D., Saito K. (2013). Effect of salt concentration of cesium solution on cesium-binding capacity of potassium cobalt-hexacyanoferrate-impregnated fiber. Kagaku Kogaku Ronbunshu.

[101] Lehto J., Harjula R. (1987). Separation of cesium from nuclear waste solutions with hexacyanoferrate(II)s and ammonium phosphomolybdate. Solvent Extr. Ion Exch..

[102] Liang C., Liu P., Xu J., Wang H., Wang W., Fang J., Wang Q., Shen W., Zhao J. (2011). A simple method for the synthesis of Fe-Co Prussian Blue analogue with novel morphologies, different structures, and dielectric properties. Synth. React. Inorg. Met.-Org. Nano-Met. Chem..

[103] Liu H.D., Li F.Z., Zhao X., Yun G.C. (2009). Preparing high-loaded potassium cobalt hexacyanoferrate/silica composite for radioactive wastewater treatment. Nucl. Technol..

[104] Valsala T.P., Joseph A., Shah J.G., Raj K., Venugopal V. (2009). Synthesis and characterization of cobalt ferrocyanides loaded on organic anion exchanger. J. Nucl. Mater..

[105] Vo V., Van Minh N., Lee H.I., Kim J.M., Kim Y., Kim S.J. (2009). Synthesis and characterization of Co-Fe Prussian blue nanoparticles within MCM-41. Mater. Res. Bull..

[106] Li B., Liao J., Wu J., Zhang D., Zhao J., Yang Y., Cheng Q., Feng Y., Liu N. (2008). Removal of radioactive cesium from solutions by zinc ferrocyanide. Nucl. Sci. Tech..

[107] Nilchi A., Hadjmohammadi M.R., Garmarodi S.R., Saberi R. (2009). Studies on the adsorption behavior of trace amounts of (90)Sr^2+^, (140)La^3+^, (60)Co^2+^, Ni^2+^ and Zr^4+^ cations on synthesized inorganic ion exchangers. J. Hazard. Mater..

[108] Shakir K., Sohsah M., Soliman M. (2007). Removal of cesium from aqueous solutions and radioactive waste simulants by coprecipitate flotation. Sep. Purif. Technol..

[109] Chen R., Tanaka H., Kawamoto T., Asai M., Fukushima C., Na H., Kurihara M., Watanabe M., Arisaka M., Nankawa T. (2013). Selective removal of cesium ions from wastewater using copper hexacyanoferrate nanofilms in an electrochemical system. Electrochim. Acta.

[110] Egorin A.M., Avramenko V.A. (2012). Dynamics of sorption of cesium radionuclides on selective ferrocyanide sorbents. Distribution of the ^137^Cs activity in the stationary phase. Radiochemistry.

[111] Epimakhov V., Moskvin L., Chetverikov V., Epimakhov T., Ganyushkin A., Prokhorkin S. (2010). Treatment of water from spent nuclear fuel storage basins with ion-exchange resins modified with transition metal hexacyanoferrates. Radiochemistry.

[112] Harjula R., Lehto J., Paajanen A., Brodkin L., Tusa E. (2001). Removal of radioactive cesium from nuclear waste solutions with the transition metal hexacyanoferrate ion exchanger CsTreat. Nucl. Sci. Eng..

[113] Ishfaq M.M., Safdar M. (1999). A radiochemical study of the kinetics and mechanism of caesium ion adsorption on potassium copper nickel hexacyanoferrate(II). Adsorpt. Sci. Technol..

[114] Kamenik J., Dulaiova H., Sebesta F., St’astna K. (2013). Fast concentration of dissolved forms of cesium radioisotopes from large seawater samples. J. Radioanal. Nucl. Chem..

[115] Mimura H., Kimura M., Akiba K., Onodera Y. (1999). Separation of Cesium and Strontium by Potassium Nickel. J. Nucl. Sci. Technol..

[116] Peterskova M., Valderrama C., Gibert O., Cortina J.L. (2012). Extraction of valuable metal ions (Cs, Rb, Li, U) from reverse osmosis concentrate using selective sorbents. Desalination.

[117] Sheveleva I., Avramenko V., Bratskaya S., Zheleznov V., Modin E., Sergienko V. (2010). Composite sorbents for recovery of cesium radionuclides. Russ. J. Appl. Chem..

[118] Sheveleva I., Zheleznov V., Bratskaya S., Avramenko V., Kuryavyi V. (2011). Sorption of cesium radionuclides with composite carbon fibrous materials. Russ. J. Appl. Chem..

[119] Sun B., Hao X.G., Wang Z.D., Guan G.Q., Zhang Z.L., Li Y.B., Liu S.B. (2012). Separation of low concentration of cesium ion from wastewater by electrochemically switched ion exchange method: Experimental adsorption kinetics analysis. J. Hazard. Mater..

[120] Taj S., Muhammad D., Chaudhry M.A., Mazhar M. (2011). Lithium, rubidium and cesium ion removal using potassium iron(III) hexacyanoferrate(II) supported on polymethylmethacrylate. J. Radioanal. Nucl. Chem..

[121] Tsuruoka S., Fugetsu B., Khoerunnisa F., Minami D., Takeuchi K., Fujishige M., Hayashi T., Kim Y.A., Park K.C., Asai M. (2013). Intensive synergetic Cs adsorbent incorporated with polymer spongiform for scalable purification without post filtration. Mater. Express.

[122] Valsala T.P., Roy S.C., Shah J.G., Gabriel J., Raj K., Venugopal V. (2009). Removal of radioactive caesium from low level radioactive waste (LLW) streams using cobalt ferrocyanide impregnated organic anion exchanger. J. Hazard. Mater..

[123] Vipin A.K., Ling S., Fugetsu B. (2014). Sodium cobalt hexacyanoferrate encapsulated in alginate vesicle with CNT for both cesium and strontium removal. Carbohydr. Polym..

[124] Voronina A., Semenishchev V., Nogovitsyna E., Betenekov N. (2012). A study of ferrocyanide sorbents on hydrated titanium dioxide support using physicochemical methods. Radiochemistry.

[125] Voronina A.V., Semenishchev V.S. (2015). Sorption-active matrix based on titanium hydroxide for concentration and joint immobilization of caesium and strontium radionuclides. J. Radioanal. Nucl. Chem..

[126] Watari K., Imai K., Izawa M. (1968). Radiochemical application of “Iron Ferrocyanide-Anion Exchange Resin”. J. Nucl. Sci. Technol..

[127] Vashnia S., Tavakoli H., Cheraghali R., Sepehrian H. (2014). Supporting of lead hexacyanoferrate on mesoporous MCM-41 and its use as effective adsorbent for strontium: Equilibrium, kinetic, and thermodynamic studies. Sep. Sci. Technol..

[128] Gogoi D., Shanmugamani A.G., Rao S.V.S., Kumar T., Shreekumar B., Sinha P.K. (2013). Studies on adsorptive removal of radioactive cobalt from alkaline waste generated in sodium cooled fast breeder reactors. J. Radioanal. Nucl. Chem..

[129] Harish S., Joseph J., Phani K.L.N. (2011). Interaction between gold(III) chloride and potassium hexacyanoferrate(II/III)-Does it lead to gold analogue of Prussian blue?. Electrochim. Acta.

[130] Mimura H., Sakakibara T., Yan W., Niibori Y., Koyama S.I., Ohnishi T. (2011). Selective uptake of palladium from high-level liquid wastes by hybrid microcapsules enclosed with insoluble ferrocyanides. Proceedings of the 13th International Conference on Environmental Remediation and Radioactive Waste Management ICEM2010.

[131] Sheha R.R. (2012). Preparation and performance of a novel composite as a reactive resin for copper retention. Chem. Eng. J..

[132] Rykov A.I., Wang J., Zhang T., Nomura K. (2013). Cs sorption by “soluble” and “insoluble” iron hexacyanocobaltates probed by Mössbauer spectroscopy. Hyperfine Interact..

[133] Ofomaja A.E., Pholosi A., Naidoo E.B. (2014). Kinetics and competitive modeling of cesium biosorption onto iron(III) hexacyanoferrate modified pine cone powder. Int. Biodeterior. Biodegrad..

[134] Volkov A.G., Paula S., Deamer D.W. (1997). Two mechanisms of permeation of small neutral molecules and hydrated ions across phospholipid bilayers. Bioelectrochem. Bioenerg..

[135] Qing Y., Li J., Kang B., Chang S., Dai Y., Long Q., Yuan C. (2015). Selective sorption mechanism of Cs^+^ on potassium nickel hexacyanoferrate(II) compounds. J. Radioanal. Nucl. Chem..

[136] Nilchi A., Atashi H., Javid A.H., Saberi R. (2007). Preparations of PAN-based adsorbers for separation of cesium and cobalt from radioactive wastes. Appl. Radiat. Isot..

[137] Moon J.K., Lee E.H., Kim H.T. (2004). Ion exchange of Cs ion in acid solution with potassium cobalt hexacyanoferrate. Korean J. Chem. Eng..

[138] Takahatake Y., Watanabe S., Shibata A., Nomura K., Koma Y. (2012). Decontamination of radioactive liquid waste with hexacyanoferrate(II). Procedia Chemistry.

[139] Sinha P.K., Amalraj R.V., Krishnasamy V. (1993). Flocculation studies on freshly precipitated copper ferrocyanide for the removal of caesium from radioactive liquid waste. Waste Manag..

[140] Hu L., Mei J.Y., Chen Q.W., Zhang P., Yan N. (2011). Magnetically separable Prussian blue analogue Mn-3 Co(CN)(6) (2)center dot nH(2)O porous nanocubes as excellent absorbents for heavy metal ions. Nanoscale.

[141] Zhang H., Zhao X., Wei J., Li F. (2014). Sorption behavior of cesium from aqueous solution on magnetic hexacyanoferrate materials. Nucl. Eng. Des..

[142] Dashtinejad M., Samadfam M., Fasihi J., Fumeshkenar F.G., Sepehrian H. (2014). Synthesis, characterization, and cesium sorption performance of potassium nickel hexacyanoferrate-loaded granular activated carbon. Part. Sci. Technol..

[143] Liu H.-D., Li F.-Z., Zhao X. (2008). Preparation of high surface area porous potassium titanium hexacynoferrate/SiO_2_ bead for radioactive waste water treatment. Chin. J. Inorg. Chem..

[144] Folch B., Larionova J., Guari Y., Molvinger K., Luna C., Sangregorio C., Innocenti C., Caneschi A., Guerin C. (2010). Synthesis and studies of water-soluble Prussian Blue-type nanoparticles into chitosan beads. Phys. Chem. Chem. Phys..

[145] De Mattos I.L., Gorton L. (2001). Metal-hexacyanoferrate films: A tool in analytical chemistry. Quim. Nova.

[146] Kulesza P.J., Miecznikowski K., Chojak M., Malik M.A., Zamponi S., Marassi R. (2001). Electrochromic features of hybrid films composed of polyaniline and metal hexacyanoferrate. Electrochim. Acta.

[147] Narang J., Chauhan N., Pundir C.S. (2013). Construction of triglyceride biosensor based on nickel oxide-chitosan/zinc oxide/zinc hexacyanoferrate film. Int. J. Biol. Macromol..

[148] Rao S.V.S., Lal K.B., Narasimhan S.V., Ahmed J. (1999). Copper ferrocyanide-polyurethane foam as a composite ion exchanger for removal of radioactive cesium. J. Radioanal. Nucl. Chem..

[149] Rao S.V.S., Narasimhan S.V., Lal K.B. (2003). Composite CFC-PU foam ion exchanger in the removal of radioactive cesium. J. Radioanal. Nucl. Chem..

[150] Rao S.V.S., Lekshmi R., Mani A.G.S., Sinha P.K. (2010). Treatment of low level radioactive liquid wastes using composite ion-exchange resins based on polyurethane foam. J. Radioanal. Nucl. Chem..

[151] Kuang J., Yuk K.Y., Huh K.M. (2011). Polysaccharide-based superporous hydrogels with fast swelling and superabsorbent properties. Carbohydr. Polym..

[152] Vincent C., Hertz A., Vincent T., Barré Y., Guibal E. (2014). Immobilization of inorganic ion-exchanger into biopolymer foams—Application to cesium sorption. Chem. Eng. J..

[153] Vincent C., Barré Y., Vincent T., Taulemesse J.M., Robitzer M., Guibal E. (2015). Chitin-Prussian blue sponges for Cs(I) recovery: From synthesis to application in the treatment of accidental dumping of metal-bearing solutions. J. Hazard. Mater..

[154] Folch B., Guari Y., Larionova J., Luna C., Sangregorio C., Innocenti C., Caneschi A., Guerin C. (2008). Synthesis and behaviour of size controlled cyano-bridged coordination polymer nanoparticles within hybrid mesoporous silica. New J. Chem..

[155] Thammawong C., Opaprakasit P., Tangboriboonrat P., Sreearunothai P. (2013). Prussian blue-coated magnetic nanoparticles for removal of cesium from contaminated environment. J. Nanopart. Res..

[156] Lin Y., Fryxell G.E., Wu H., Engelhard M. (2001). Selective sorption of cesium using self-assembled monolayers on mesoporous supports. Environ. Sci. Technol..

[157] Voronina A.V., Semenishchev V.S. (2013). Effect of surface modification of hydrated titanium dioxide on its selectivity to strontium. Radiochemistry.

[158] Sharygin L.M., Muromskii A.Y. (2000). New inorganic sorbent for ion-selective purification of liquid radioactive wastes. At. Energ..

[159] Sharygin L.M., Muromskii A.Y. (2004). Inorganic sorbent for selective treatment of liquid radioactive wastes. Radiochemistry.

[160] Jeerage K.M., Schwartz D.T. (2000). Characterization of cathodically deposited nickel hexacyanoferrate for electrochemically switched ion exchange. Sep. Sci. Technol..

[161] Ding D., Lei Z., Yang Y., Feng C., Zhang Z. (2014). Selective removal of cesium from aqueous solutions with nickel(II) hexacyanoferrate(III) functionalized agricultural residue-walnut shell. J. Hazard. Mater..

[162] Causse J., Tokarev A., Ravaux J., Moloney M., Barre Y., Grandjean A. (2014). Facile one-pot synthesis of copper hexacyanoferrate nanoparticle functionalised silica monoliths for the selective entrapment of Cs-137. J. Mater. Chem. A.

[163] Yi K., Jin R.G. (2012). Study on optimum coagulation conditions of high molecular weight PAN fiber in wet spinning by orthogonal experimental design. Fibers Polym..

[164] Vincent T., Taulemesse J.M., Dauvergne A., Chanut T., Testa F., Guibal E. (2013). Thallium(I) sorption using prussian blue immobilized in alginate capsules. Carbohydr. Polym..

[165] Nilchi A., Malek B., Ghanadi Maragheh M., Khanchi A. (2003). Exchange properties of cyanide complexes. J. Radioanal. Nucl. Chem..

[166] Loos-Neskovic C., Abousahl S., Fedoroff M. (1990). Column-usable inorganic fixator preparation by localized growth on a solid alkaline ferrocyanide. J. Mater. Sci..

[167] Hu M., Torad N.L.K., Chiang Y.-D., Wu K.C.W., Yamauchi Y. (2012). Size- and shape-controlled synthesis of Prussian Blue nanoparticles by a polyvinylpyrrolidone-assisted crystallization process. Crystengcomm.

[168] Andersen T., Melvik J.E., Gasered O., Alsberg E., Christensen B.E. (2012). Ionically gelled alginate foams: Physical properties controlled by operational and macromolecular parameters. Biomacromolecules.

[169] Galambos M., Dano M., Rosskopfova O., Sersen F., Kufcakova J., Adamcova R., Rajec P. (2012). Effect of gamma-irradiation on adsorption properties of Slovak bentonites. J. Radioanal. Nucl. Chem..

[170] Ghosh S.K., Chaki T.K., Khastgir D., Pinto R. (2015). Gamma Irradiation effects on optical, thermal, and mechanical properties of polysulfone/MWCNT nanocomposites in argon atmosphere. J. Appl. Polym. Sci..

[171] Islam M.M., Khan M.A., Rahman M.M. (2015). Preparation of gelatin based porous biocomposite for bone tissue engineering and evaluation of gamma irradiation effect on its properties. Mater. Sci. Eng. C.

[172] Devi P.S.R., Bhatt H., Deo M.N., Verma R., Reddy A.V.R. (2014). Effect of gamma irradiation on the ion exchange capacity of polyaniline. Radiat. Phys. Chem..

[173] Olatunji M.A., Khandaker M.U., Mahmud H., Amin Y.M. (2015). Influence of adsorption parameters on cesium uptake from aqueous solutions- a brief review. RSC Adv..

[174] Thallapally P.K., Motkuri R.K., Fernandez C.A., McGrail B.P., Behrooz G.S. (2010). Prussian Blue analogues for CO_2_ and SO_2_ capture and separation applications. Inorg. Chem..

[175] Chelebaeva E., Larionova J., Guari Y., Ferreira R.A.S., Carlos L.D., Trifonov A.A., Kalaivani T., Lascialfari A., Guerin C., Molvinger K. (2011). Nanoscale coordination polymers exhibiting luminescence properties and NMR relaxivity. Nanoscale.

[176] Long J., Vallat R., Ferreira R.A.S., Carlos L.D., Almeida Paz F.A., Guari Y., Larionova J. (2012). A bifunctional luminescent single-ion magnet: Towards correlation between luminescence studies and magnetic slow relaxation processes. Chem. Commun..

