# Supplementary material for: Immobilization of Metal Hexacyanoferrate Ion-Exchangers for the Synthesis of Metal Ion Sorbents—A Mini-Review
Source: Molecules. 2015 Nov 19;20(11):20582–613. doi: 10.3390/molecules201119718 (PMC6332395; doi:10.3390/molecules201119718)
Supplement: Supplementary file 1 [file molecules-20-19718-s001.pdf]

Supplementary Materials: Immobilization of Metal Hexacyanoferrate Ion-Exchangers for the Synthesis of Metal Ion Sorbents-A Mini-Review

Thierry Vincent, Chloë Vincent and Eric Guibal

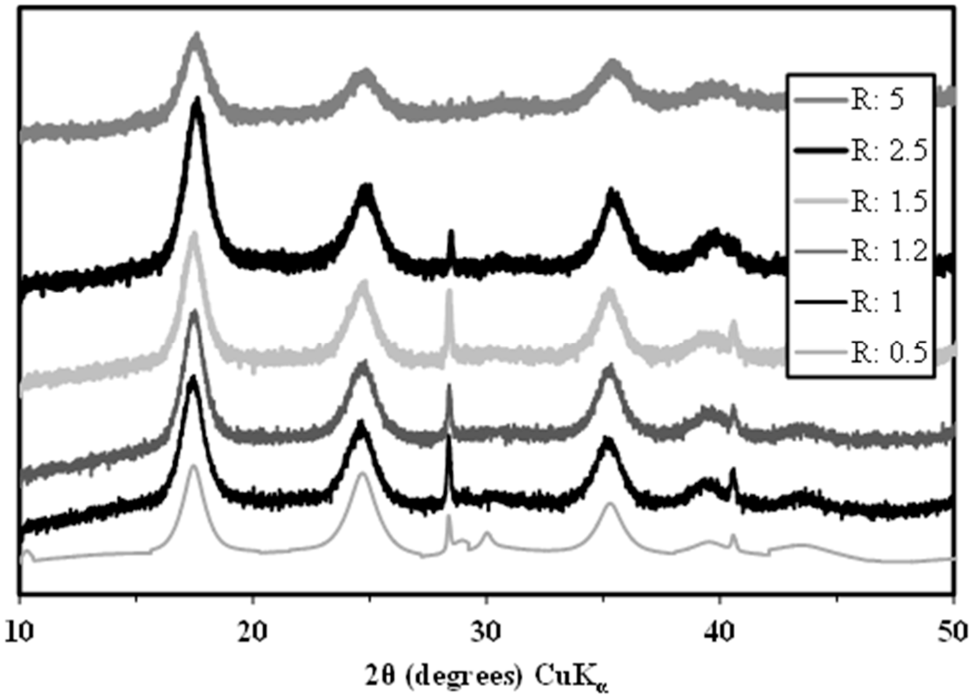

Figure S1. Effect of R (molar ratio between the precursors, R = $[FeCl_3]/[K_4Fe(CN)_6]$ on X-ray diffraction patterns of Prussian Blue, in-drop synthesis).

Table S1. Textural and chemical characteristics of metal hexacyanoferrate/chitin beads prepared with different metal precursors [18].

Type	K Content (mmol·K·g⁻¹)	Met. Content (mmol·M·g⁻¹)	Fe Content (mmol·Fe·g⁻¹)	Ion-Exch. Content (%, *w/w*)	$K_d \times 10^5$ (mL·g⁻¹) *	Cs Sorption Cap. (mg·Cs·g⁻¹) **
HCFe-Ni	0.8	1.0	0.6	20	3.5	101.4 [461]
HCFe-Zn	0.5	0.8	0.6	16	1.6	96.4 [507)
HCFe-Co	0.9	0.7	0.5	20	33	80.7 [425]
HCFe-Cu	0.5	0.9	0.5	18	19	117.9 [621]
HCFe-Fe	0.2	-	1.6	16	10	42.4 [236]

*: Distribution coefficient determined on ^{137}Cs synthetic solutions (initial activity: 37,000 Bq L^{-1}) [18].
**: Into brackets: the sorption capacities reported in function of the actual amount of ion-exchanger in the composite.

Table S2. XRD analysis – Peak positions and crystallite size (calculated using the Scherrer equation) (Into parenthesis: standard deviation; (Average peak positions (2θ): 17.42 ± 0.11 (200) − 24.63 ± 0.12 (220) − 35.22 ± 0.16 (400)).

R	Peak Position (2θ)	Crystallite Size (Å)	Average Crystallite Size (Å)
0.5	17.445	80	71 (7)
	24.670	62	
	35.263	72	
1.0	17.397	65	103 (32)
	24.596	101	
	34.886	144	
1.2	17.220	93	82 (8)
	24.396	76	
	35.231	78	
1.5	17.427	80	78 (2)
	24.621	78	
	35.202	75	
2.5	17.556	80	80 (2)
	24.779	77	
	35.402	82	
5.0	17.504	61	58 (8)
	24.698	67	
	35.342	47	

Table S3. SEM-EDX analysis of PB compressed-discs.

R	C	N	Cl	K	Fe	Fe/K
0.5	41.68 (0.14)	38.30 (0.38)	2.12 (0.12)	10.67 (0.13)	7.23 (0.01)	0.68
1	40.12 (0.11)	41.36 (0.06)	1.78 (0.01)	8.61 (0.08)	8.14 (0.04)	0.94
1.2	40.09 (0.08)	41.73 (0.09)	2.46 (0.02)	7.43 (0.06)	8.29 (0.06)	1.12
1.5	40.04 (0.34)	40.81 (0.43)	2.22 (0.045)	8.44 (0.03)	8.50 (0.02)	1.00
2.5	39.33 (0.28)	44.96 (0.51)	2.73 (0.07)	3.50 (0.05)	9.49 (0.13)	2.72
5	38.32 (0.06)	43.74 (0.5)	5.51 (0.04)	2.52 (0.02)	9.91 (0.06)	3.94

R = $[FeCl_3]/[K_4Fe(CN)_6]$: molar ratio of precursors used for the synthesis of PB composites; Into parenthesis: standard deviation.

Table S4. Metals used for synthesizing hexacyanoferrate-based sorbents.

Metal	Ni	Cu	Fe	Co	Zn
References	[6–8,12, 28,30–66]	[8,11,13,22,25,28,30,31,38,42, 45–49,58,61,64,65,67–81]	[26–28,42,61, 79,81–96]	[1,8,9,23,28,30,31,42, 45–48,58,75,88,97–105]	[14,45–48, 50,106–108]

Table S5. Target metals for sorption on hexacyanoferrate-based sorbents.

Metal	References
Cs	[1,7,8,12,17,22,23,27,30–34,36–38,43, 45–47,51–53,55–58,62,63,65,67–71,74,75, 77–81,83,86,87,89,92–94,96,98–100,103,108–126]
Rb	[54,116,120]
Tl	[95]
Sr	[43,91,107,123,127]
Co	[39,54,107,128]
Au	[129]
Cr	[44,54] *
As	[44] *
Pd	[49,130]
Ag	[50]
Pb	[73]
Li	[116,120]
I	[64]
La	[107]
U	[116]
Sb	[54]
Cu	[131]

*: metal sorption after complexation with a ligand.
